# Supplementary material for: Graphlet eigencentralities capture novel central roles of genes in pathways
Source: PLoS One. 2022 Jan 25;17(1):e0261676. doi: 10.1371/journal.pone.0261676 (PMC8789115; doi:10.1371/journal.pone.0261676)
Supplement: S1 File — (PDF) [file pone.0261676.s001.pdf]

Supplementary materials: “Graphlet eigencentralities capture novel central roles of genes in pathways”

Sam F. L. Windels, Noël Malod-Dognin, and Nataša Pržulj

Contents

1 Data statistics **4**
1.1 Basic network statistics . . . 4
1.2 The number of pathways considered per molecular network . . 4
1.3 The distribution of pathway sizes per molecular network . . . 4

2 Methodology **5**
2.1 Centrality measures . . . 5
2.1.1 Degree centrality . . . 5
2.1.2 Graphlet centrality . . . 6
2.1.3 Core number . . . 6
2.1.4 Betweenness centrality . . . 6
2.1.5 Closeness centrality . . . 7
2.1.6 Eccentricity . . . 7
2.2 Set enrichment analysis . . . 7
2.2.1 Assigning ancestor annotations to pathways . . . 8
2.2.2 Assigning GO-term annotations to pathways . . . 8
2.2.3 Pathway set enrichment . . . 8
2.3 Random model network generation . . . 9
2.4 Network distance measures . . . 9
2.4.1 Graphlet based network distance . . . 9
2.4.2 Non-graphlet based network distance measures . . . 10

3 Comparing graphlet eigencentrality to other node centralities. **10**
3.1 Comparison of different node centralities in model networks . . 10
3.2 Comparison of different node centralities in molecular networks 19

4 Graphlet adjacencies describe topologically and biologically distinct pathways **26**
4.1 Pathway participation prediction accuracy . . . 27
4.2 Identifying pathways described by graphlet adjacencies . . . 32
4.3 Graphlet adjacencies describe complementary groups of functionally related pathways . . . 38
4.4 Pathways described by the same graphlet adjacency are topologically similar . . . 45
4.5 Linking pathways described by graphlet adjacencies to model networks . . . 50

5 Graphlet eigencentralities capture complementary cancer mechanisms 52
5.1 Cancer related gene prediction accuracy 52
5.2 The number of cancer genes predicted and their overlap 56

1 Data statistics

1.1 Basic network statistics

	Nodes	Density	Diam.
**PPI yeast**	5,881	0.0055	6
**PPI human**	17,380	0.0019	9
**COEX yeast**	5,363	0.0129	4
**COEX human**	15,373	0.0131	4
**GI yeast**	5,634	0.0273	6

Supplementary Table 1: **Network statistics.** The columns 'nodes', 'Density' and 'Diameter' respectively report the number of nodes, density and diameter of each of the molecular networks (first column).

1.2 The number of pathways considered per molecular network

	Nr. of pathways
PPI yeast All	187
PPI human All	969
PPI human Disease	92
PPI human Non-disease	877
COEX yeast All	141
COEX human All	712
COEX human Disease	68
COEX human Non-disease	644
GI yeast All	241

Supplementary Table 2: **Number of pathways considered for each molecular network.**

1.3 The distribution of pathway sizes per molecular network

We create a set of pathway networks for each of our five molecular networks by inducing the gene set of each pathway in Reactome on the full molecular network (see Section 2.4.2 of the main paper). In Supplementary Figure 1, we provide the distribution of pathway sizes for each of our molecular networks.

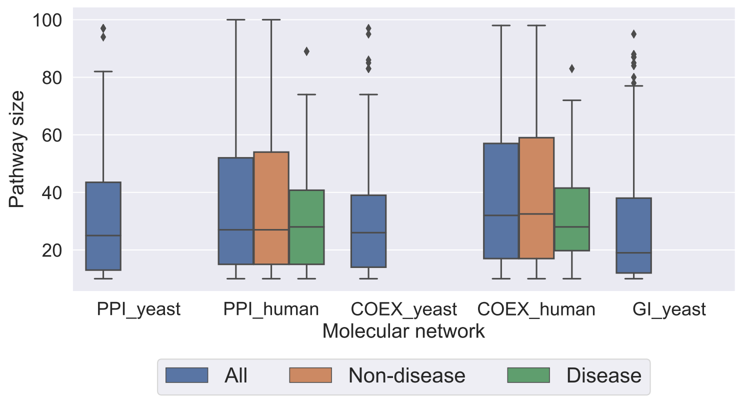

Supplementary Figure 1: **Distribution of pathway sizes per molecular network.** Each box plot represents the distribution of the pathway sizes for each of our molecular networks (x-axis) considering all pathways, non-disease pathways and disease pathways (colour) in the Reactome ontology.

2 Methodology

2.1 Centrality measures

Network centrality measures quantify the importance of a node in a network based on some topological property. We consider the formal definition of eigencentrality and extend this definition to graphlet eigencentrality in Sections 2.1 and 2.2 of the main paper. Here, we define a selection of node centrality measures applied in network biology. For more details, we refer the reader to Newman (2010).

2.1.1 Degree centrality

The *degree centrality* considers highly connected nodes to be the most important nodes in the network. The degree centrality of a node is synonymous with its degree: it is its number of neighbours in the network, or equivalently, the number of edges in the network including the node. Formally, the degree centrality of a node, $u \in V$, is:

$$DC(u) = d_u^{G_0} = \sum_{v=1}^{n} A_{uv}^{G_0}, \tag{1}$$

where $d_u^{G_0}$ is the number of times node u touches graphlet 0 (i.e. an edge), n is the number of nodes in the network and $A_{uv}^{G_0}$ is the graphlet adjacency

matrix for graphlet 0 (equivalent to the standard adjacency matrix). It was shown that perturbing nodes with a high degree in PPI networks has a higher probability of impacting cell viability (Jeong *et al.*, 2001).

2.1.2 Graphlet centrality

The *graphlet centrality* of a node, $u \in V$, is the weighted sum of its log transformed graphlet degrees:

$$GCD(u) = \sum_{i=0}^{8} w_i \times log(d_u^{G_i} + 1), \tag{2}$$

where w_i is a weight coefficient to take into account redundancies between graphlet counts and the log transformations scales graphlet counts for different graphlets to the same order of magnitude (Milenković *et al.*, 2011). The graphlet centrality is a direct extension of the degree centrality, designed to take the extended neighbourhood of a node into account. For instance, a node with a low degree that touches many of the 4-node graphlets would rank higher when considering its graphlet centrality instead of its degree centrality. Thus, GDC captures the wider impact of a node on the network. Based on this idea, it was shown that GDC can be used to uncover disease genes and drug targets in the human PPI network (Milenković *et al.*, 2011)..

2.1.3 Core number

The k*-core* of a network is the maximal subgraph such that all nodes in the subgraph have a degree of at least k. The *core number* of a node u is c if the node is in the k-core for $k = c$ and not in the k-core for $k = c + 1$. Nodes with a high core number in the human PPI network have been shown to likely be part of the *core diseaseome*, a subnetwork of the PPI network of statistically significantly similarly wired and functionally similar disease genes (Janjić and Pržulj, 2012).

2.1.4 Betweenness centrality

The *betweenness centrality* measures the amount of control u has on the flow of information in the network. Formally, the betweenness centrality of a node u is the fraction of shortest paths between all nodes in the network on which u occurs over all shortest paths in the network:

$$BC(u) = \sum^{s,t \in V} \frac{\sigma(s, t|u)}{\sigma(s, t)}, \tag{3}$$

where $\sigma(s,t)$ is the number of shortest paths in the network from node s to node t, and $\sigma(s,t|v)$ is the number of those paths that include node u. As the betweenness centrality captures direct and indirect influences of proteins distant in the network, the betweenness centrality indicates how important a node is within the wider context of the network. It was shown in the yeast PPI network that genes with a high betweenness, regardless of their degree, are likely to be essential genes. The authors explain this through their observation that many genes with high betweenness also have a low degree, and therefore function as hub nodes linking functional modules within the PPI network (Joy *et al.*, 2005).

2.1.5 Closeness centrality

The *closeness centrality* considers a node to be central in the network if it is nearby to all other nodes in the network. Formally, the closeness centrality a node, $u \in V$, is equal to the reciprocal of the average distance of u to every other node in the network:

$$CC(u) = \frac{1}{\sum_{v=1}^{n} d(u,v)}, \tag{4}$$

were $d(u,v)$ is the shortest path distance between u and v.

2.1.6 Eccentricity

The *eccentricity* of a node in the network measures how distant it is from any other node in the network. Formally, the eccentricity of a node, $u \in V$, is equal to the longest shortest path distance from u to any of the nodes in the network:

$$ECC(u) = max_{v \in V}\{d(u,v)\}, \tag{5}$$

where $d(u,v)$ is the shortest path distance from node u to v. Thus, a node is considered important in the network, i.e. central, if it has low eccentricity.

2.2 Set enrichment analysis

In Section 3.1.2 of the main paper, we investigate if a set of pathways contains similar types of pathways and biological functions. We first annotate all pathways with their second level *ancestors*, i.e., annotations in the second most general level of the pathway ontology, and annotate each pathway with the GO-term annotations in which its respective gene set is enriched. Then, we apply pathway-set enrichment analysis to check if a set of pathways is enriched in pathways sharing pathway ancestry or GO-term annotations.

2.2.1 Assigning ancestor annotations to pathways

The Reactome Ontology is a collection of 23 direct acyclic graphs (dags), where nodes represent pathway annotations and directed edges represent 'is a'- relationships. We annotate each pathway with its ancestor terms found 1 step away from the root node of the corresponding dag. That is, to annotate a given pathway with its ancestor(s), we first find that pathway in the Reactome dag, from there trace the Reactome Ontology dag upwards (against the direction of the 'is_a' relationships) until we reach the pathway annotation(s) that is(are) one step away from the root node(s), and use the annotations corresponding to these nodes as ancestor annotations.

2.2.2 Assigning GO-term annotations to pathways

We annotate each pathway with the GO-terms in which its respective gene set is enriched, considering GO biological process terms (GO-BP), GO cellular component terms (GO-CC) and GO molecular function terms (GO-MF). To determine the enriched annotations, we apply classical gene set enrichment analysis, where we consider a set of genes as a 'sampling without replacement' experiment counting each time we find a given GO-term annotation as a 'success'. The probability of observing the same or higher enrichment (i.e. successes) of the given GO-term annotation purely by chance is equal to:

$$p = 1 - \sum_{i=0}^{X-1} \binom{K}{i} \binom{M-K}{N-i} / \binom{M}{N}, \tag{6}$$

where N is the number of GO-term annotated genes in the pathway, X is the number of genes annotated with the given GO-term annotation in the pathway, M is the number of GO-term annotated genes covered by all pathways, and K is the number of genes annotated with the given GO-term annotation over all pathways. A GO-term annotation is considered to be statistically significantly enriched if its enrichment p-value is lower than or equal to 5% after application of the Benjamini and Hochberg correction for multiple hypothesis testing.

2.2.3 Pathway set enrichment

To assess a set of pathways is statistically significantly enriched by pathways sharing ancestor annotations or GO-term annotations, we apply the hypergeometric test. That is, we consider a set of pathways as a 'sampling without replacement' experiment, in which each time we find a given ancestor or GO-term annotation, we count that as a 'success'.

The probability of observing the same or higher enrichment (i.e. successes) of the given annotation purely by chance is equal to:

$$p = 1 - \sum_{i=0}^{X-1} \binom{K}{i} \binom{M-K}{N-i} / \binom{M}{N}. \tag{7}$$

where N is the number of GO-term or ancestor annotated pathways in the pathway-set, X is the number of pathways annotated with the given ancestor or GO-term annotation in the pathway, M is the number of ancestor or GO-term annotated pathways pathways and K is the number of pathways annotated with the given ancestor or GO-term annotation over all pathways in the pathway-set. An ancestor or GO-term annotation is considered to be statistically significantly enriched if its enrichment p-value is lower than or equal to 5% after application of the Benjamini and Hochberg correction for multiple hypothesis testing.

2.3 Random model network generation

For each pathway, as induced on each of the molecular networks, we generate ten networks containing the same number of nodes and edges, for each of the following seven random network models: Erdős-Rènyi random graphs (ER) (Erdős Paul and Rényi Alfréd, 1959), generalized random graphs with the degree distribution matching to the input graph (ER-DD) (Newman, 2010), Barabási-Albert scale-free networks (SF) (Barabási and Albert, 1999), geometric random graphs (GEO) (Penrose, 2003), geometric graphs that model gene duplications and mutations (GEO-GD) (Pržulj *et al.*, 2010), stickiness-index based networks (Sticky) (Pržulj and Higham, 2006), nonuniform PSO graphs (nPSO) (Muscoloni and Cannistraci, 2018). We provide a summary on the basic properties of these networks and how to generate in (Windels *et al.*, 2019).

2.4 Network distance measures

To measure the topological dissimilarity between different sets of pathways and model networks, we consider graphlet-based and non-graphlet based network distance measures.

2.4.1 Graphlet based network distance

The Graphlet Correlation Distance (GCD-11) is the current state of the art heuristic for measuring the topological distance between networks (Yaveroğlu

et al., 2014, 2015). First, the global wiring pattern of a network is captured in its Graphlet Correlation Matrix (GCM), an 11×11 symmetric matrix comprising the pairwise Spearman's correlations between 11 different graphlet based counts over all nodes in the network. The Graphlet Correlation Distance between two networks is computed as the Euclidean distance of the upper triangle values of their GCMs.

2.4.2 Non-graphlet based network distance measures

The difference between the following non-graphlet based network descriptors can be used to measure the distance between two networks:

- The *degree distribution* is the distribution of node degrees over all nodes. It is summarised as a vector of counts, i.e. the kth value is the number of nodes that have degree k. To measure the distance between two networks, this vector is first rescaled to reduce the contribution of higher degree nodes. The pairwise distance between two networks is the euclidean distance between their rescaled degree distribution vectors. For more details, see (Yaveroğlu *et al.*, 2014).
- The *average clustering coefficient* is the total number of three node cliques in the network over the number of possible three node cliques in the network. The distance between two networks is the absolute difference of their average clustering coefficient.

3 Comparing graphlet eigencentrality to other node centralities.

Here we investigate the agreement/relationship between our new graphlet eigencentrality measures and state of the art centrality measures used in network biology in a selection of well investigated model networks and our set of molecular networks. For a review of current centrality measures, see Landherr *et al.* (2010).

3.1 Comparison of different node centralities in model networks

For each type of model network (see Supplementary Section 2.3) we generate ten networks and over the nodes of those networks, we compute the average pairwise Spearman correlation between any two different node centrality

measures (see Section 2.1). Results are presented in Supplementary Figures 2 to 9. We exclude graphlet eigencentrality for graphlet G_8 from our comparisons in ER, GeoGD, SF and SFGD networks, as this graphlet rarely occurs in them.

First of all, we observe across all model networks that most centrality measures are positively correlated. The exceptions are the clustering coefficient and eccentricity, which are typically anti-correlated to the other centrality measures. In the case of eccentricity, this is expected from its very definition, as the more eccentric a node is, the less important it is expected to be in the network. The clustering coefficient does not correlate with other centrality measures as, unlike most centrality measures, the local density of the network does not affect it. For instance, a node that is part of a triangle but with no other connections to the network would have a high clustering coefficient of 1.0 (the maximal score) but a low degree-centrality (since it only touches 2 nodes).

Secondly, we observe that the correlations between the different centrality methods heavily depend on the model network. For instance, betweenness centrality and graphlet eigencentrality for graphlet 0 strongly correlate in Scale-Free networks, with an average correlation over ten runs of 97% (least significant p-value measured over ten runs: 0.0). On the other hand, in Geometric networks, these two centrality measures are poorly correlated, with an average correlation of only 24% (although this is still a statistically significant correlation, with the least significant p-value measured at 1.97e-19). As a consequence, the clusters of highly correlated centrality measures depend on the model network considered. For instance, in Geometric model networks, we observe clear (overlapping) clusters of highly correlated centrality measures. If we ignore the clustering coefficient and eccentricity measures, the average correlation between the different centrality measures in geometric networks is 63%. Graphlet eigencentrality for graphlets 0, 1, 2, 6, 8 and the degree centrality form a cluster of highly related centralities in Geometric networks, with an average correlation of 83%. Similarly, graphlet eigencentralities for graphlets 1, 3, 4, 5, 6, 7, the degree centrality and the graphlet centrality form a cluster of highly correlated centrality measures with an average correlation of 85%. This clustering structure is not at all present in SFGD networks, where all centrality measures, again ignoring the anti-correlated eccentricity and clustering coefficient, are highly positively correlated with an average correlation of 90%, so that no clustering of centrality measures shows.

We conclude: different graphlet eigencentralities are positively correlated with each other and most existing centrality measures. The correlations and clustering between different centrality measures is strongly depended on the

topology of the network considered.

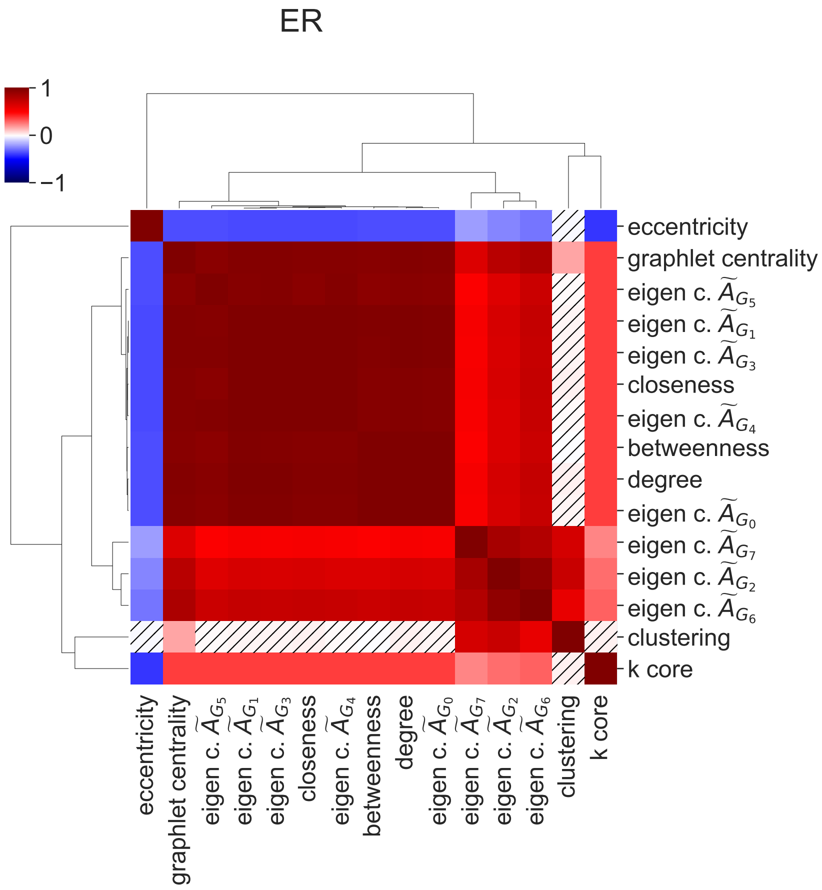

Supplementary Figure 2: **Clustered heat map of average pairwise correlations between different centrality measures over 10 ER networks.** Correlations that are not consistently significant at the 5% significance level across the 10 networks are hatched (//).

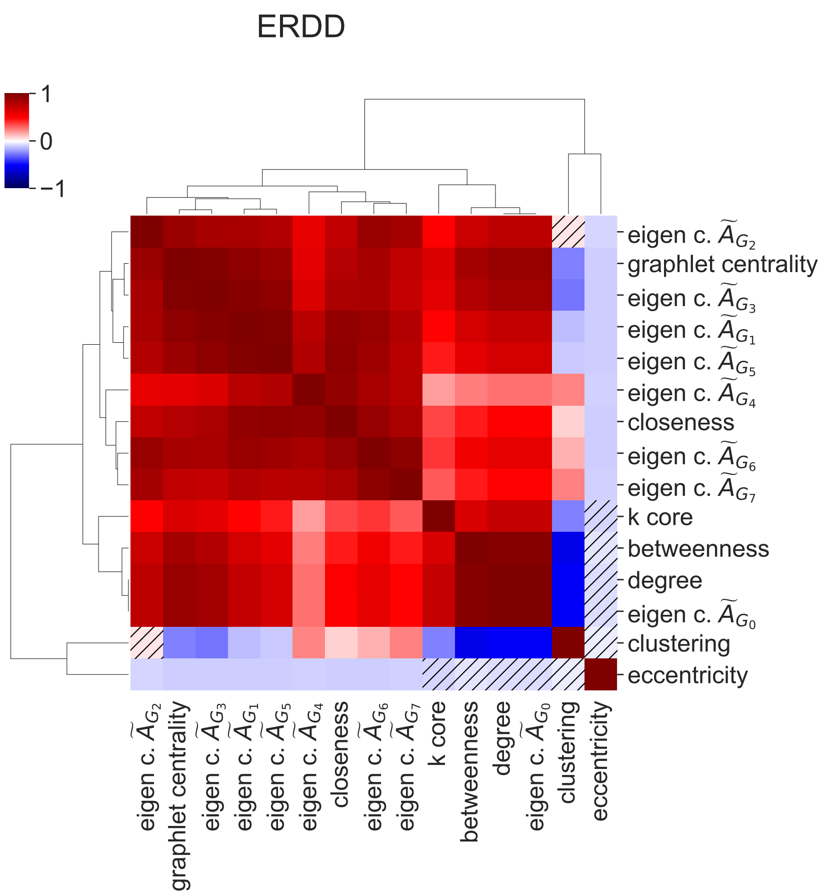

Supplementary Figure 3: **Clustered heat map of average pairwise correlations between different centrality measures over 10 ER-DD networks.** Correlations that are not consistently significant at the 5% significance level across the 10 networks are hatched (//).

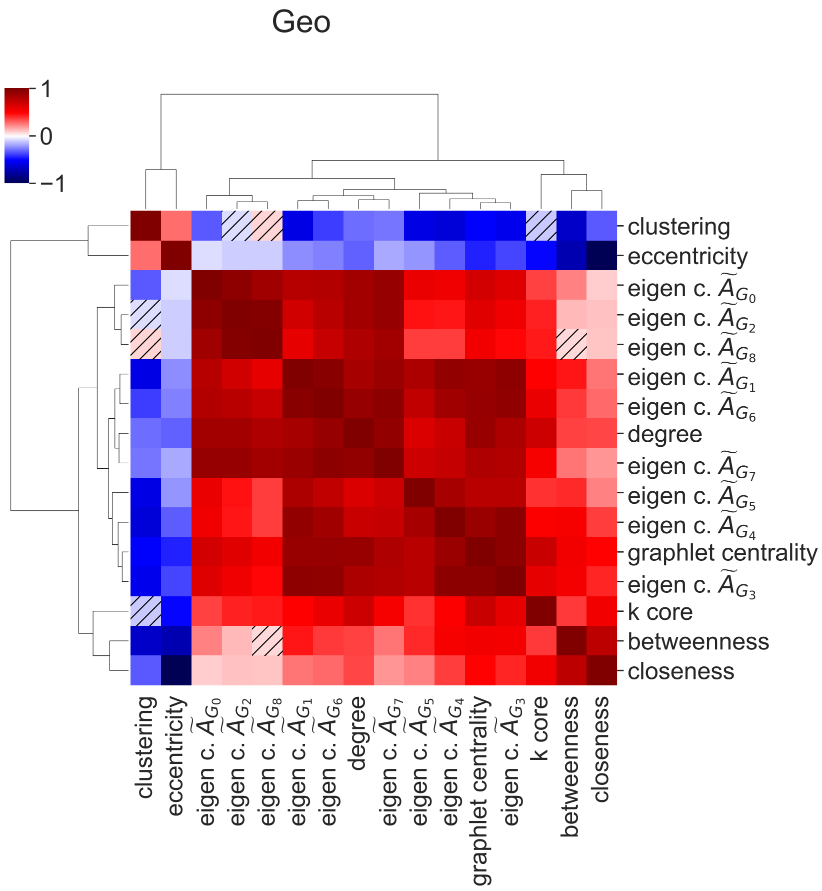

Supplementary Figure 4: **Clustered heat map of average pairwise correlations between different centrality measures over 10 Geo networks.** Correlations that are not consistently significant at the 5% significance level across the 10 networks are hatched (//).

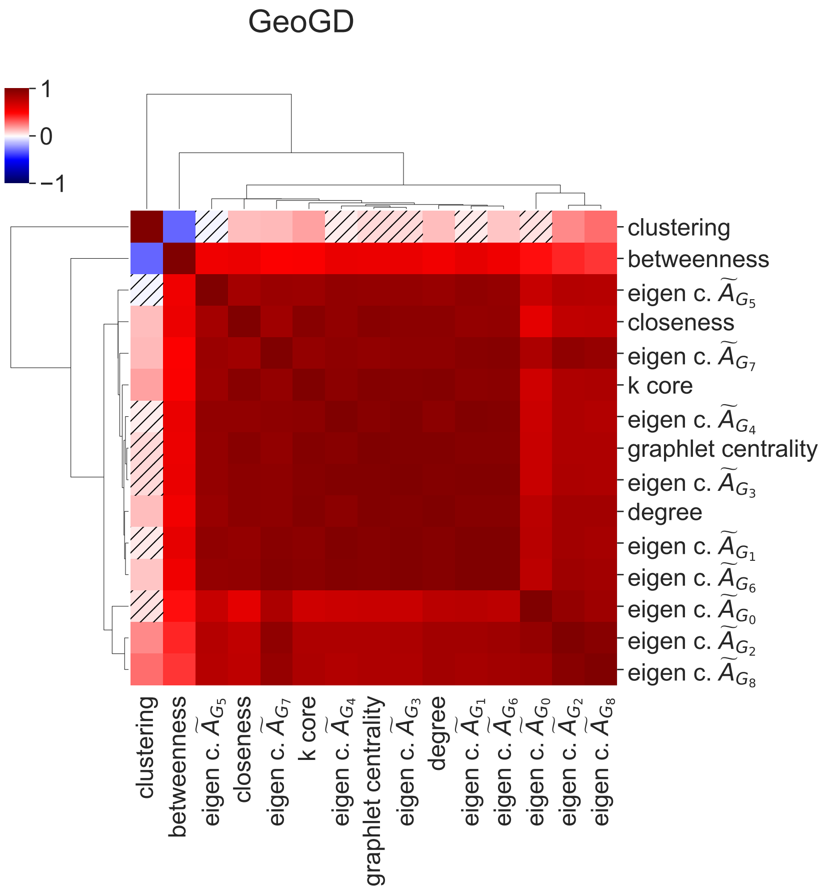

Supplementary Figure 5: **Clustered heat map of average pairwise correlations between different centrality measures over 10 GeoGD networks.** Correlations that are not consistently significant at the 5% significance level across the 10 networks are hatched (//).

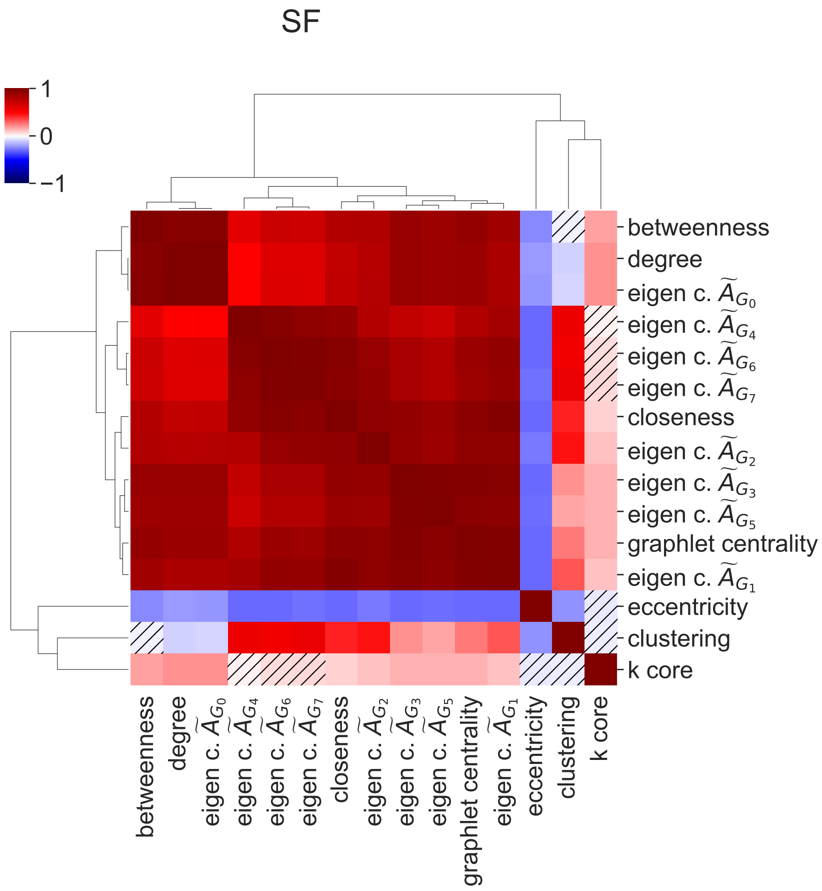

Supplementary Figure 6: **Clustered heat map of average pairwise correlations between different centrality measures over 10 SF networks.** Correlations that are not consistently significant at the 5% significance level across the 10 networks are hatched (//).

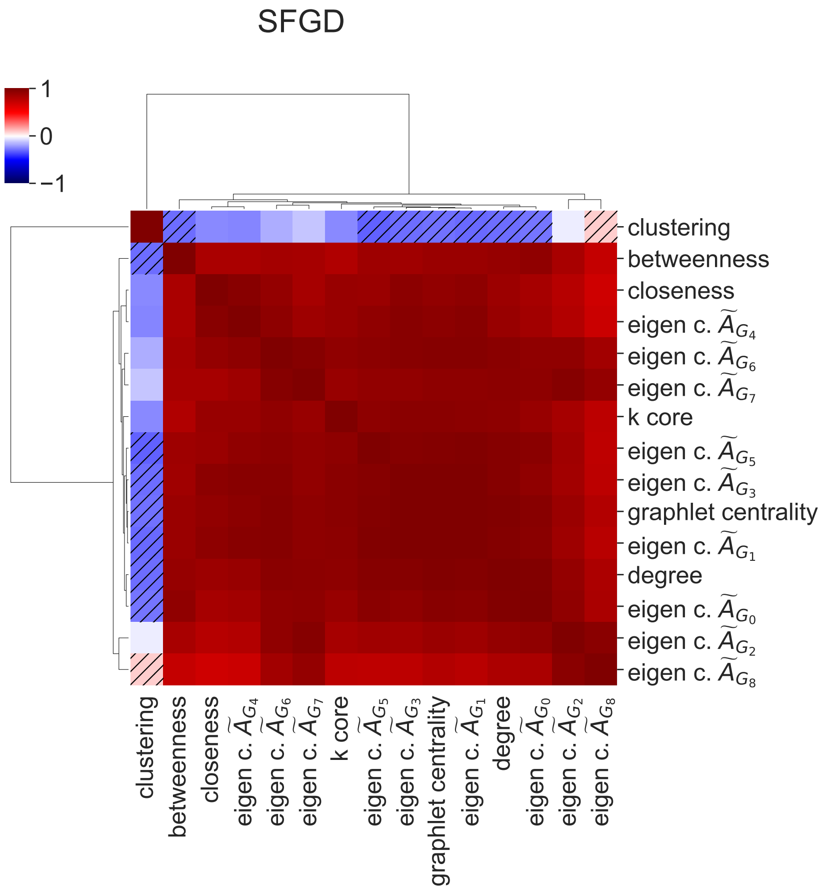

Supplementary Figure 7: **Clustered heat map of average pairwise correlations between different centrality measures over 10 SFGD networks.** Correlations that are not consistently significant at the 5% significance level across the 10 networks are hatched (//).

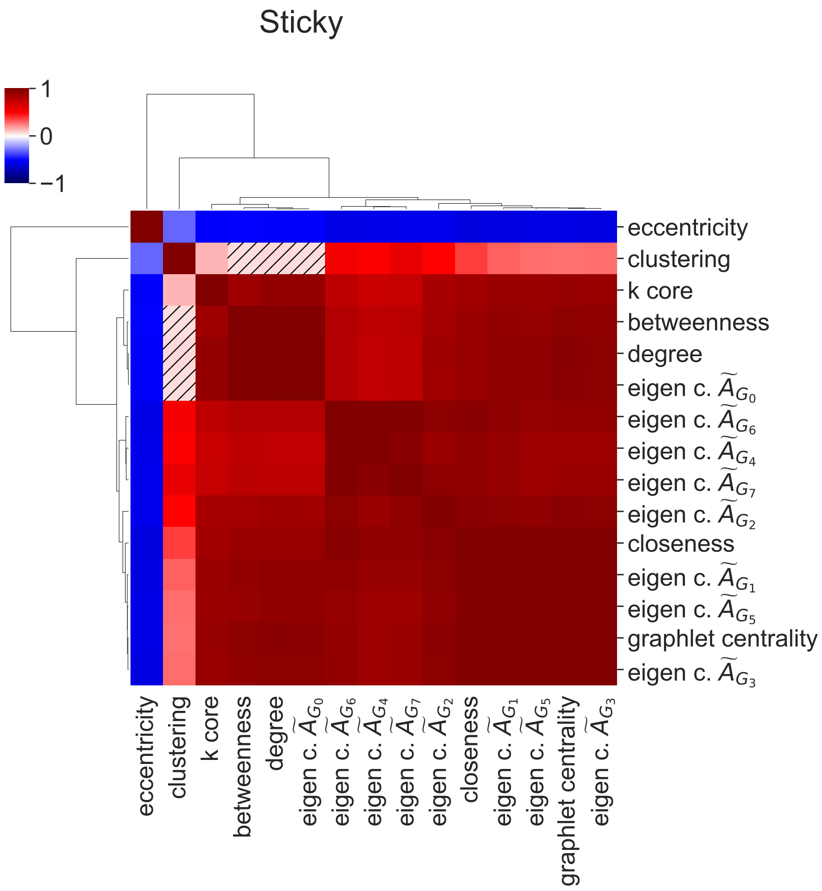

Supplementary Figure 8: **Clustered heat map of average pairwise correlations between different centrality measures over 10 Sticky networks.** Correlations that are not consistently significant at the 5% significance level across the 10 networks are hatched (//).

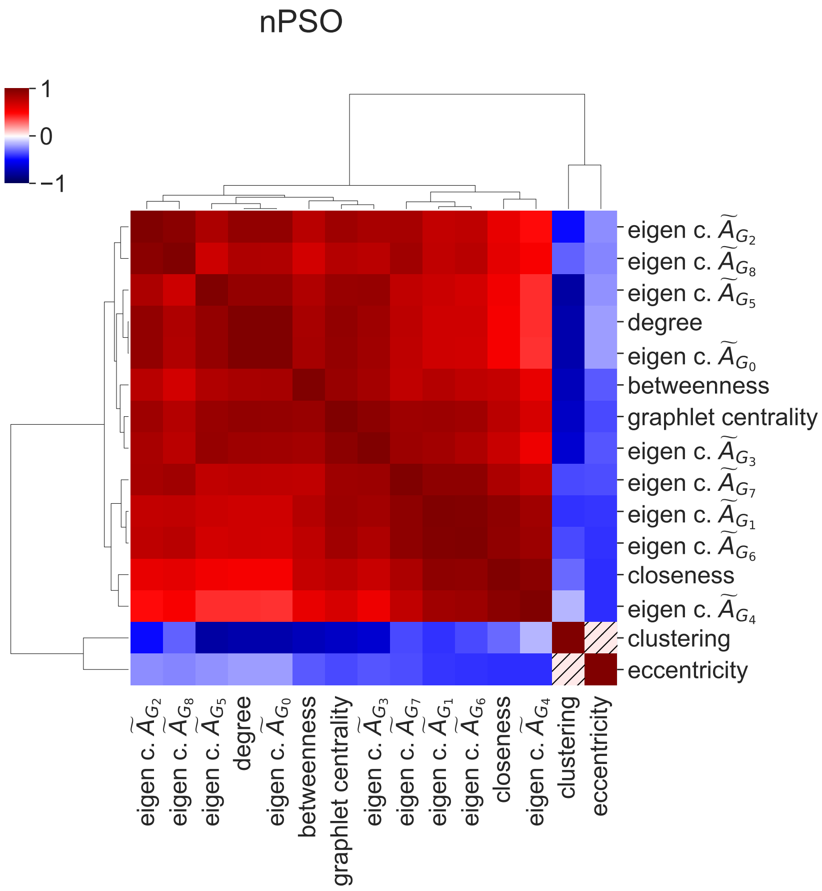

Supplementary Figure 9: **Clustered heat map of average pairwise correlations between different centrality measures over 10 nPSO networks.** Correlations that are not consistently significant at the 5% significance level across the 10 networks are hatched (//).

3.2 Comparison of different node centralities in molecular networks

For each of our molecular networks (see Section 2.7.1 in the main paper) we compute the pairwise Spearman correlation matrix between the different node centrality measures (see Supplementary Section 2.1). Results are presented in Supplementary Figures 10 to 14.

Firstly, we observe that we can make the same observations as in model networks: apart from node eccentricity and the clustering coefficient, all other node centrality methods are positively correlated. Additionally, the strength of these correlations and their clustering is dependent on the network

considered.

Secondly, we observe that graphlet eigencentralities correlate strongly. These relatively high correlations do not mean different graphlet adjacencies do not capture different biology however, as indicated by our results presented in the main paper. To provide insight into why this is the case, we present a scatter plot between the rank of the graphlet eigencentrality scores for graphlet G_0 and G_1 in the human PPI network in Supplementary Figure 15. These two eigencentralities are highly correlated, with a Spearman correlation of 86%. In the COEX networks of yeast and human, we observe that the same centrality measures cluster together. This is not true for the yeast and human PPI network. For instance, the strongest clustering in the yeast PPI network is found between the graphlet eigencentralities for graphlet 0, 2, 3, and 8, with an average correlation of 97%. The same centralities are not clustered together in the yeast PPI network and have a lower average correlation of 88%.

Thirdly, despite graphlet eigencentralities being highly correlated, visual inspection shows that a pair of highly correlated graphlet eigencentralities can agree on what nodes are very central and not at all, but still show a clear visual disagreement about the importance of the nodes not on those extremes of the centrality spectrum. To quantify this result, we measure average node overlap using the Jaccard index between the top 100 most central nodes according to the nine different graphlet eigencentralities in each of our molecular networks (see Supplementary Table 3). We find that the average Jaccard Index ranges from 0.30 in the Human COEX network to 0.76 in the yeast GI network, revealing that for all of our molecular networks, there is some disagreement between the different eigencentralities on what nodes are the most central, indicating their potential complementarity in biological applications.

We conclude: as was shown in model networks, graphlet eigencentralities cluster positively with existing centrality measures in molecular networks, with the strength of the correlations and their clustering being strongly depended on the network considered. Additionally, despite high overall agreement between the different graphlet-eigencentralities on the centrality of nodes, we show there is a distinct disagreement on the top 100 most central nodes, indicating their potential complementarity in biological applications.

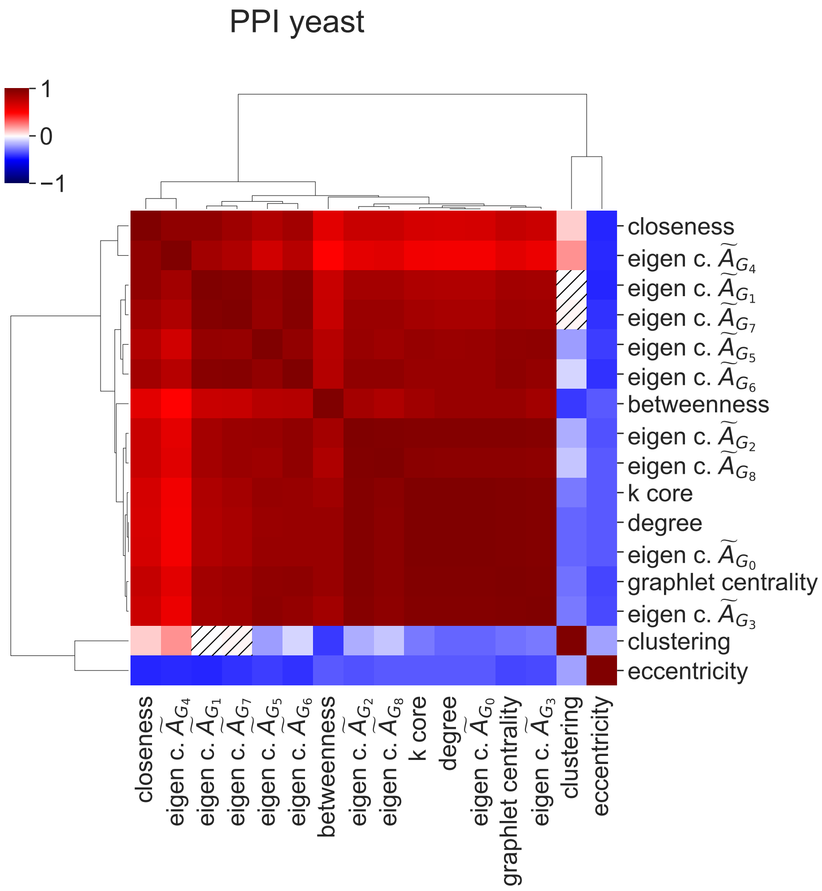

Supplementary Figure 10: **Clustered heat map of pairwise correlations between different centrality measures in the yeast PPI network.** Correlations that are not significant at the 5% significance level are hatched (//).

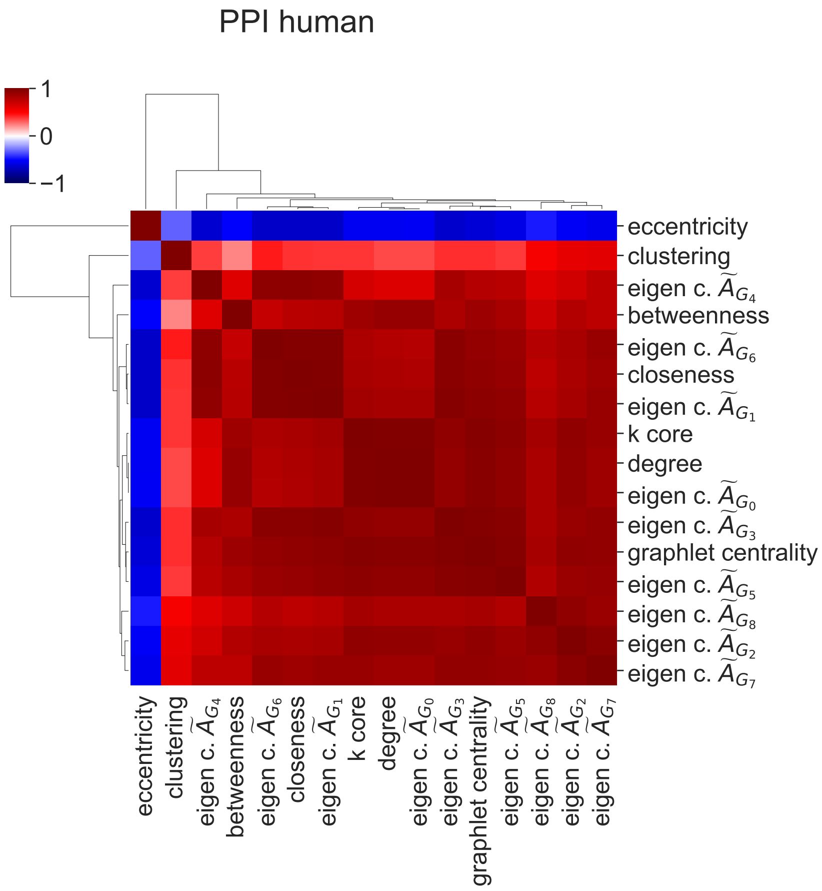

Supplementary Figure 11: **Clustered heat map of pairwise correlations between different centrality measures in the human PPI network.** Correlations that are not significant at the 5% significance level are hatched (//).

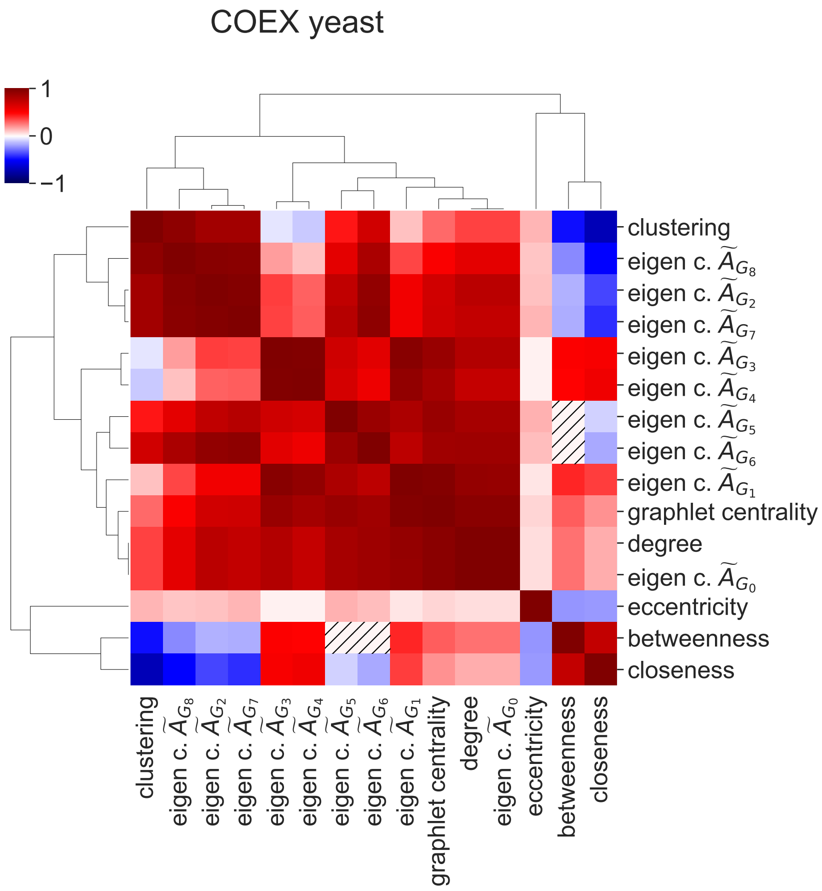

Supplementary Figure 12: **Clustered heat map of pairwise correlations between different centrality measures in the yeast COEX network.** Correlations that are not significant at the 5% significance level are hatched (//).

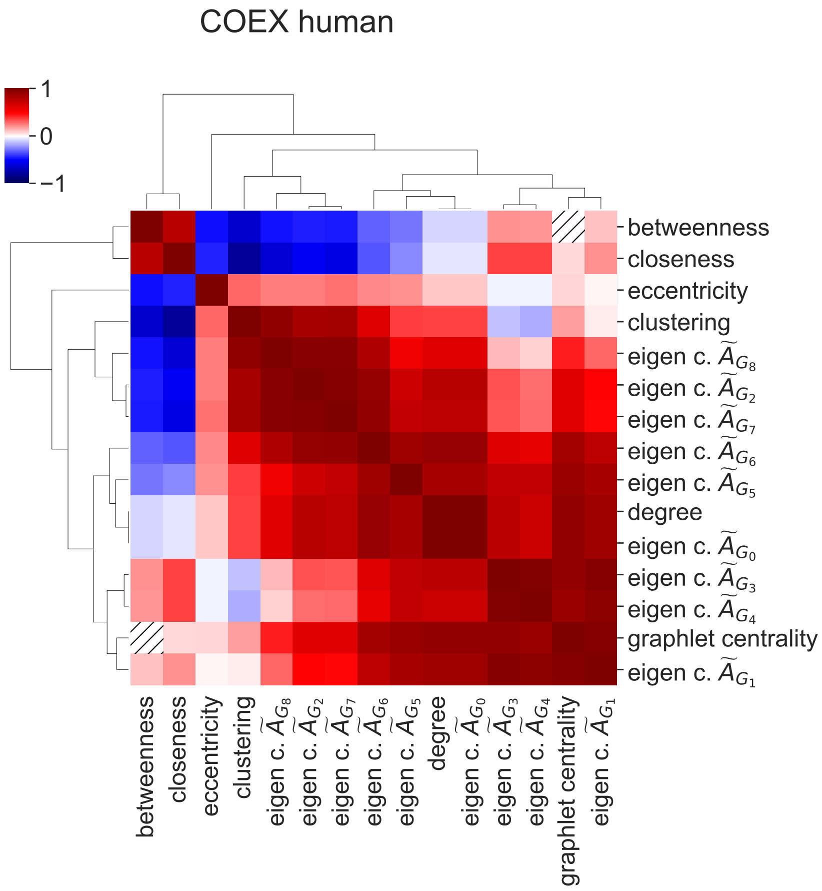

Supplementary Figure 13: **Clustered heat map of pairwise correlations between different centrality measures in the human COEX network.** Correlations that are not significant at the 5% significance level are hatched (//).

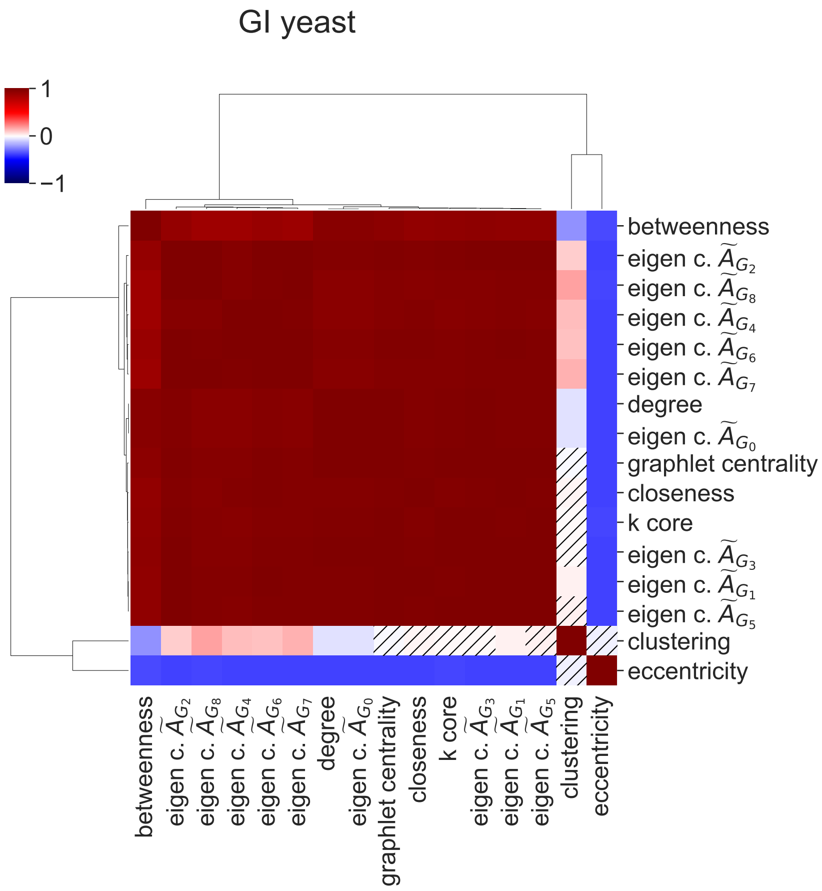

Supplementary Figure 14: **Clustered heat map of pairwise correlations between different centrality measures in the yeast GI network.** Correlations that are not significant at the 5% significance level are hatched (//).

	Average Jaccard Index	Stdev. of Jaccard Index
PPI yeast	0.65	0.19
PPI human	0.73	0.10
COEX yeast	0.31	0.23
COEX human	0.30	0.23
GI yeast	0.76	0.14

Supplementary Table 3: **Average node overlap (Jaccard index) and its standard deviation between the top 100 most central nodes according to the nine different graphlet eigencentralities in each of our molecular networks.**

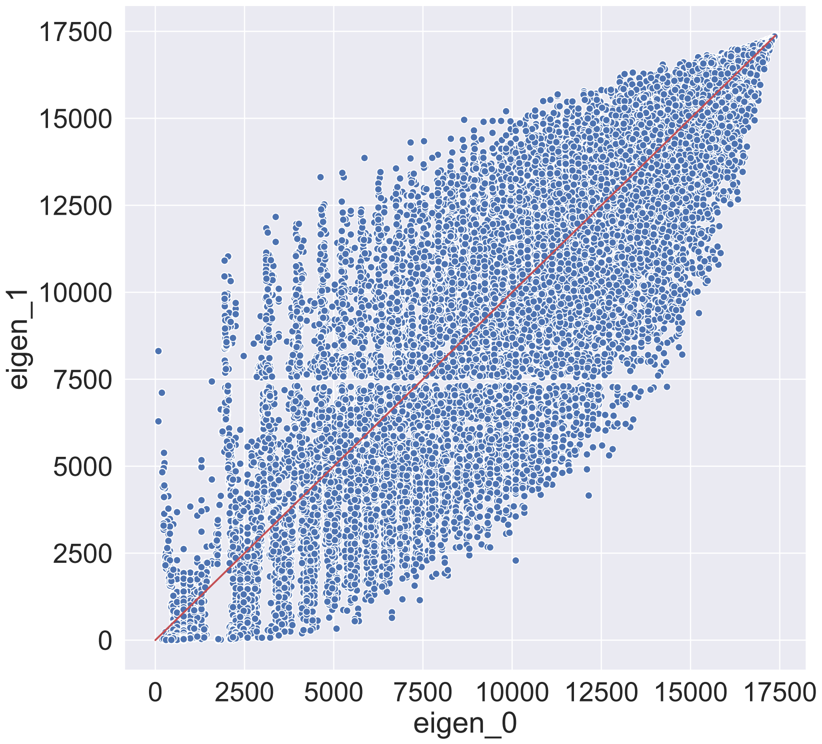

Supplementary Figure 15: **Scatter plot of the rank of graphlet eigencentrality scores for graphlet 0 and 1 (x-axis, y-axis respectively) in the human PPI network.**

4 Graphlet adjacencies describe topologically and biologically distinct pathways

To enable our investigation of topology and biology captured by different graphlet adjacencies, we first identify sets of pathways that are described by each graphlet adjacency. Per graphlet adjacency, we consider the described pathways to be those pathways for which we achieve a normalised AUC-PR higher than 3.0 (for details, see Section 3.1.1 of the main paper). We report the pathway participation prediction accuracy based on different graphlet adjacencies for our five different molecular networks in Supplementary Section 4.1. Based on these prediction accuracy results, we identify the sets of pathways described by each graphlet adjacency for each of our five molecular networks in Supplementary Section 4.2.

4.1 Pathway participation prediction accuracy

In Supplementary Figures 16 to 20, we compare the pathway participation prediction accuracy based on different graphlet adjacencies and prediction methods, for our five molecular networks.

In Supplementary Figures 16-A to 20-A, we observe that regardless of the underlying graphlet adjacency and molecular network type, our local approach and GeneMANIA consistently perform better than random (AUC-ROC=0.5), achieving median AUC-ROC scores higher than 0.6. This, except in the yeast GI network, where GeneMANIA performs close to random and in the yeast PPI network, where our local approach performs close to random when applied on graphlet adjacency $\widetilde{A_{G_4}}$. Our global approach performs as by random when applied on graphlet adjacencies for $\widetilde{A_{G_1}}$, $\widetilde{A_{G_2}}$ and $\widetilde{A_{G_8}}$ in PPI and GI networks, with median AUC-ROC scores around 0.5.

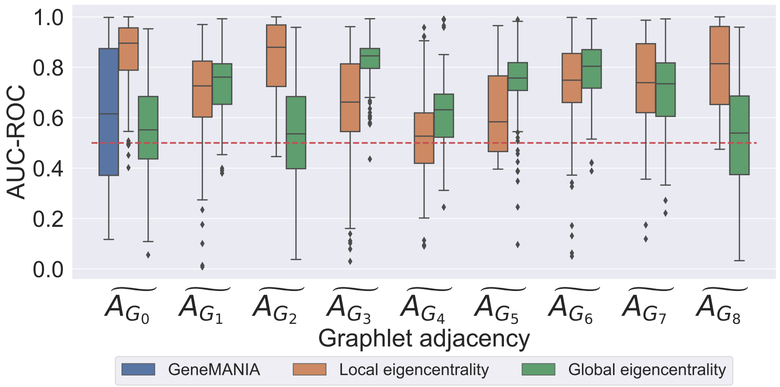

(A)

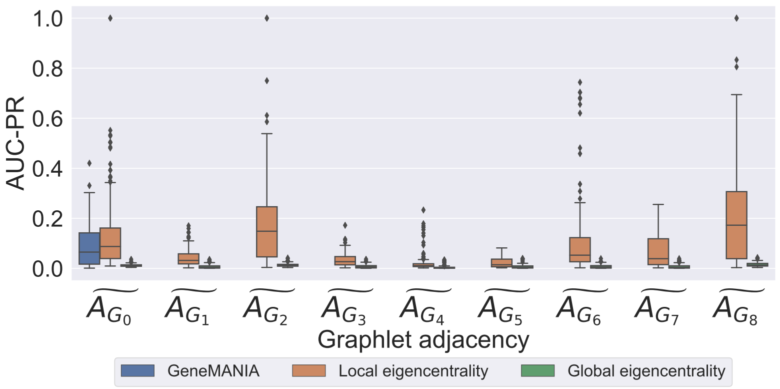

(B)

Supplementary Figure 16: **Pathway participation prediction accuracy in the yeast PPI network.** Plot (A) and (B) show the pathway participation prediction accuracy measured using AUC-ROC and AUC-PR respectively, for three methods (see legend), applied on different graphlet adjacencies (x-axis), in the yeast PPI network. Each box plot represents the distribution of prediction accuracies over all pathways using the indicated method and graphlet adjacency.

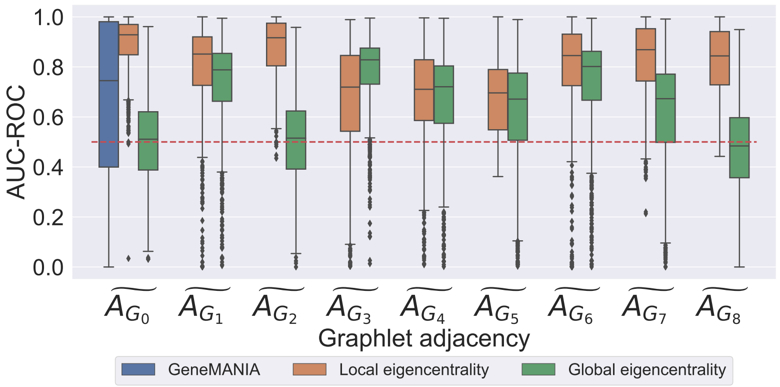

(A)

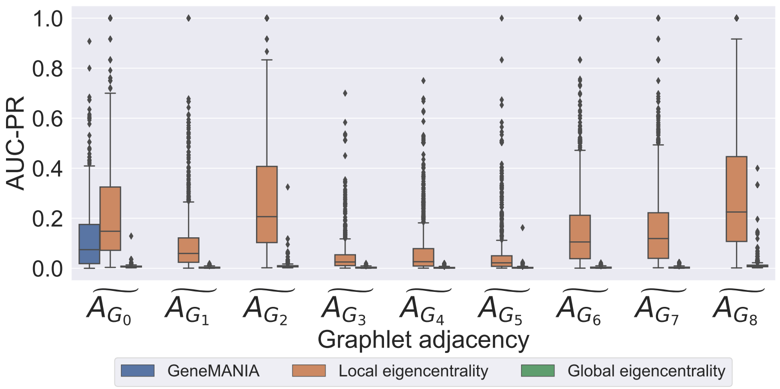

(B)

Supplementary Figure 17: **Pathway participation prediction accuracy in the human PPI network.** Plot (A) and (B) show the pathway participation prediction accuracy measured using AUC-ROC and AUC-PR respectively, for three methods (see legend), applied on different graphlet adjacencies (x-axis), in the human PPI network. Each box plot represents the distribution of prediction accuracies over all pathways using the indicated method and graphlet adjacency.

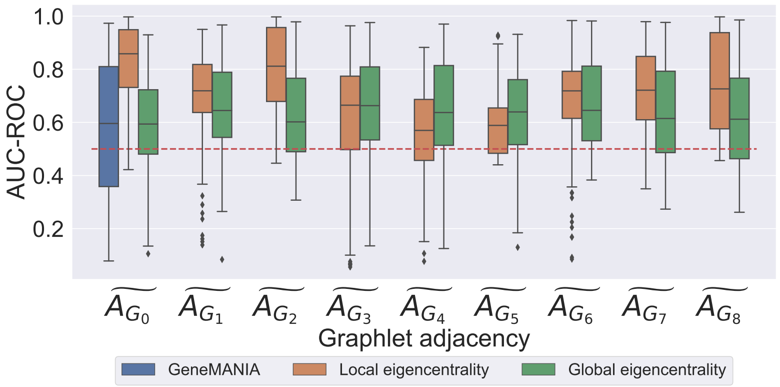

(A)

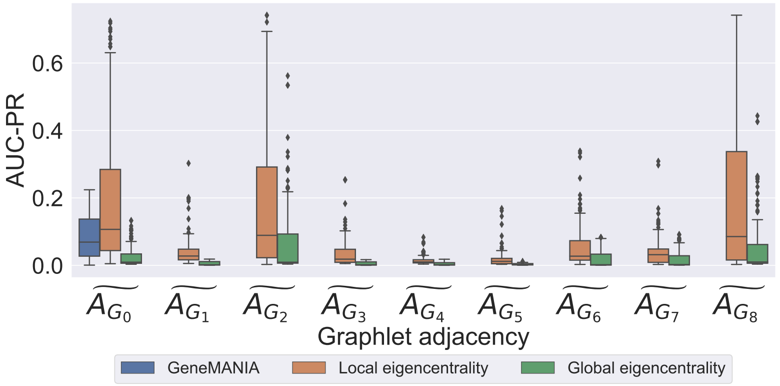

(B)

Supplementary Figure 18: **Pathway participation prediction accuracy in the yeast COEX network.** Plot (A) and (B) show the pathway participation prediction accuracy measured using AUC-ROC and AUC-PR respectively, for three methods (see legend) applied on different graphlet adjacencies (x-axis), in the yeast COEX network. Each box plot represents the distribution of prediction accuracies over all pathways using the indicated method and graphlet adjacency.

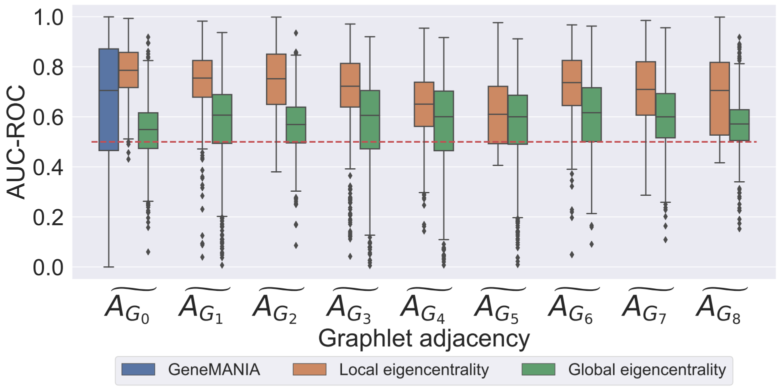

(A)

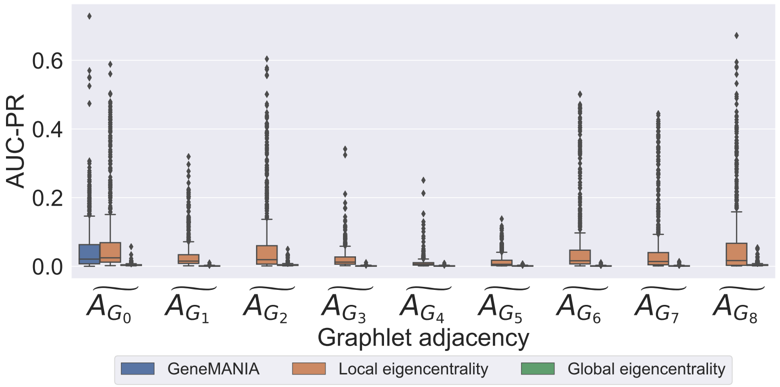

(B)

Supplementary Figure 19: **Pathway participation prediction accuracy in the human COEX network.** Plot (A) and (B) show the pathway participation prediction accuracy measured using AUC-ROC and AUC-PR respectively, for three methods (see legend) applied on different graphlet adjacencies (x-axis), in the human COEX network. Each box plot represents the distribution of prediction accuracies over all pathways using the indicated method and graphlet adjacency.

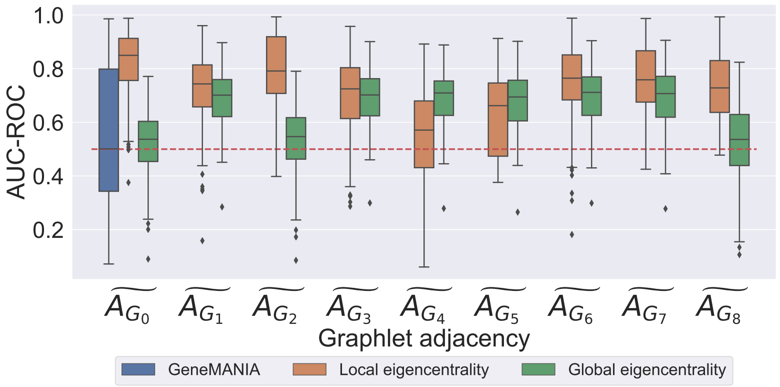

(A)

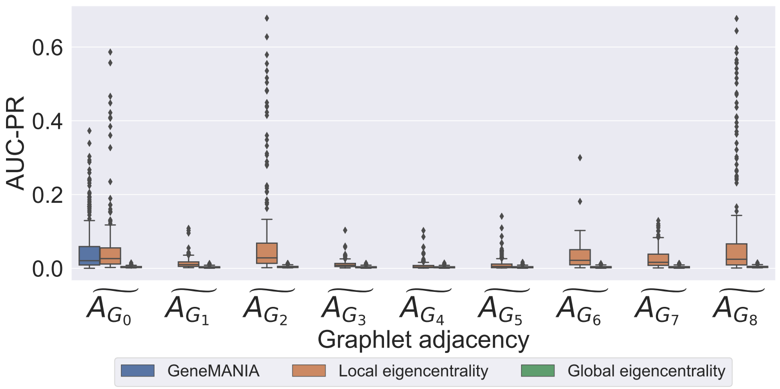

(B)

Supplementary Figure 20: **Pathway participation prediction accuracy in the yeast GI network.** Plot (A) and (B) show the pathway participation prediction accuracy measured using AUC-ROC and AUC-PR respectively, for three methods (see legend) applied on different graphlet adjacencies (x-axis), in the yeast GI network. Each box plot represents the distribution of prediction accuracies over all pathways using the indicated method and graphlet adjacency.

4.2 Identifying pathways described by graphlet adjacencies

Here we identify the pathways described by different graphlet adjacencies in our five molecular networks, based on the pathway participation prediction accuracy scores achieved using local graphlet eigencentralities. We consider the pathways described by a given graphlet adjacency to be those for which

we achieve a normalized AUC-PR score higher than 3 (in analogy to the 99.7% confidence interval of standard normally distributed variables). Here, we report the number of described pathways per type of graphlet adjacency and the overlap between the set of pathways described by different graphlet adjacencies, for our five molecular networks, in Supplementary Figures 21 to 25.

We observe in each of our molecular networks that all graphlet adjacencies describe at least some pathways. Specifically, depending on the underlying graphlet adjacency, we are able to identify between 5 to 13 pathways with a described graphlet adjacency topology in the yeast PPI network (see the bar chart in Supplementary Figure 21), between 43 and 75 pathways in the human PPI network (see the bar chart in Supplementary Figure 22), between 5 and 16 pathways in the yeast COEX network (see the bar chart in Supplementary Figure 23), between 27 and 70 pathways in the human COEX network (see the bar chart in Supplementary Figure 23) and between 5 and 22 pathways in the yeast GI network (see the bar chart in Supplementary Figure 25).

Additionally, we find that their is only little overlap between the sets of pathways described by different graphlet adjacencies within the same molecular network (as measured using the average Jaccard index). This means that different graphlet adjacencies tend to describe different pathways. Specifically, the average Jaccard Index between pathways described by different graphlet adjacencies is 0.12 in the yeast PPI network (see the heat map in Supplementary Figure 21), 0.18 in the human PPI network (see the heat map in Supplementary Figure 22),0.11 in the yeast COEX network (see the heat map in Supplementary Figure 23), 0.30 in the human network (see the heat map in Supplementary Figure 24) and 0.17 in the yeast GI network (see the heat map in Supplementary Figure 25). We investigate if the sets of pathways described by different graphlet adjacencies are actually biologically functionally different in the next section.

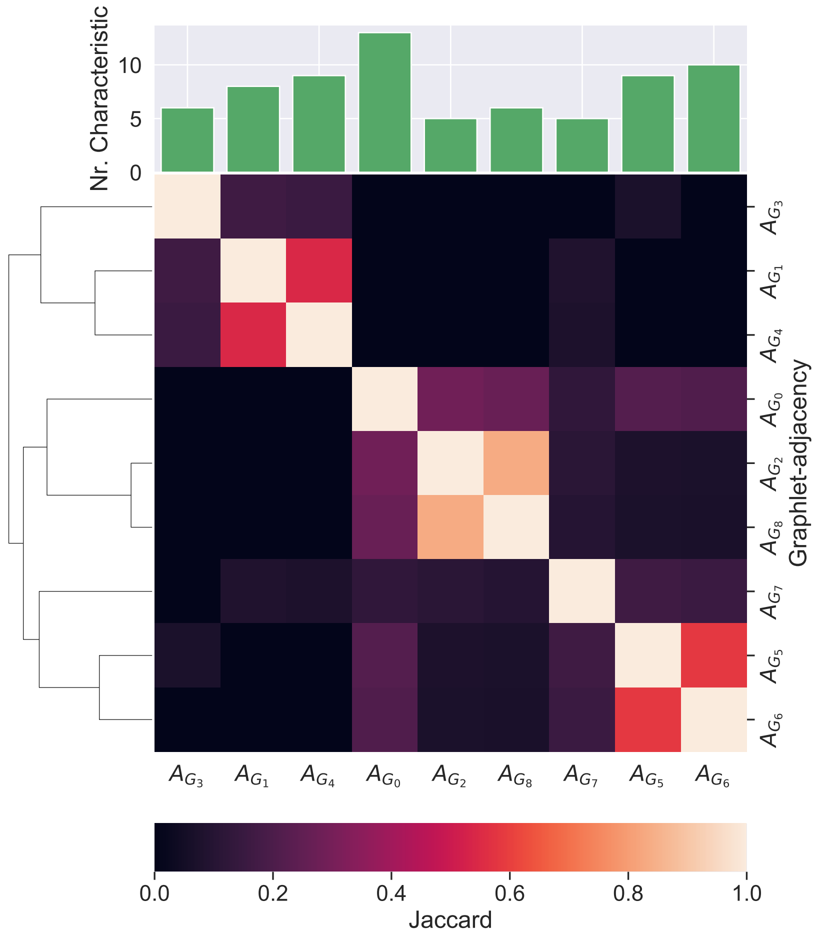

Supplementary Figure 21: **The number of pathways described by each graphlet adjacency and their overlap in the yeast PPI network.** A clustered heat map of the Jaccard similarity indices between the sets of pathways described by different graphlet adjacencies (x-axis). On top, a bar-chart indicating the number pathways described by each corresponding graphlet adjacency.

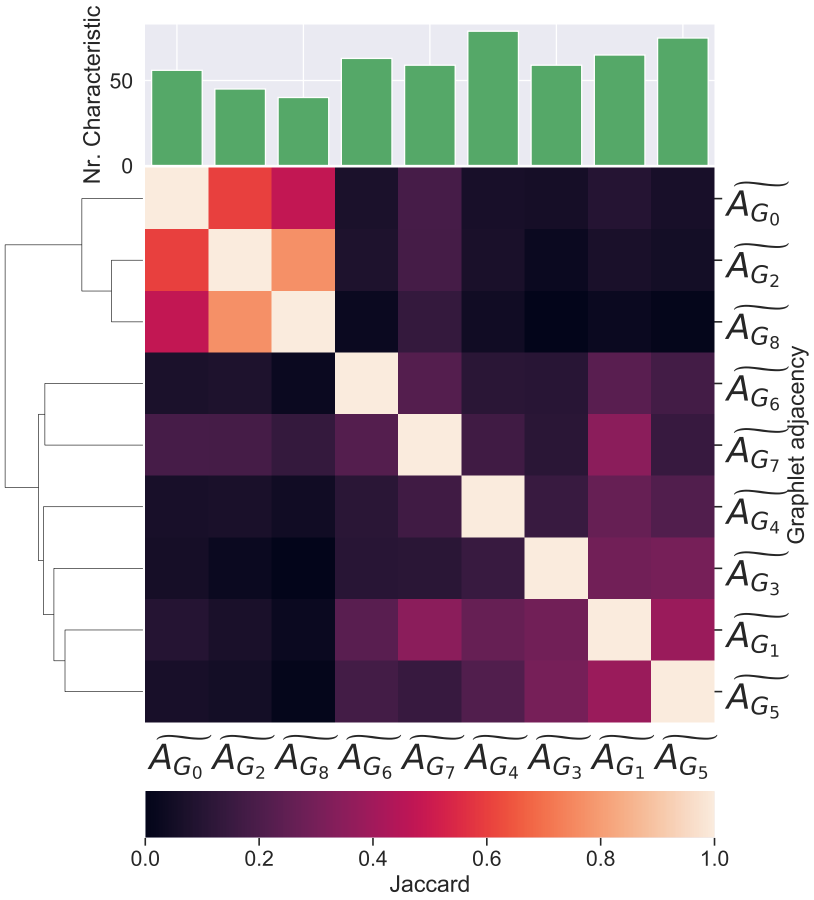

Supplementary Figure 22: **The number of pathways described by each graphlet adjacency and their overlap in the human PPI network.** A clustered heat map of the Jaccard similarity indices between the sets of pathways described by different graphlet adjacencies (x-axis). On top, a bar-chart indicating the number pathways described by each corresponding graphlet adjacency.

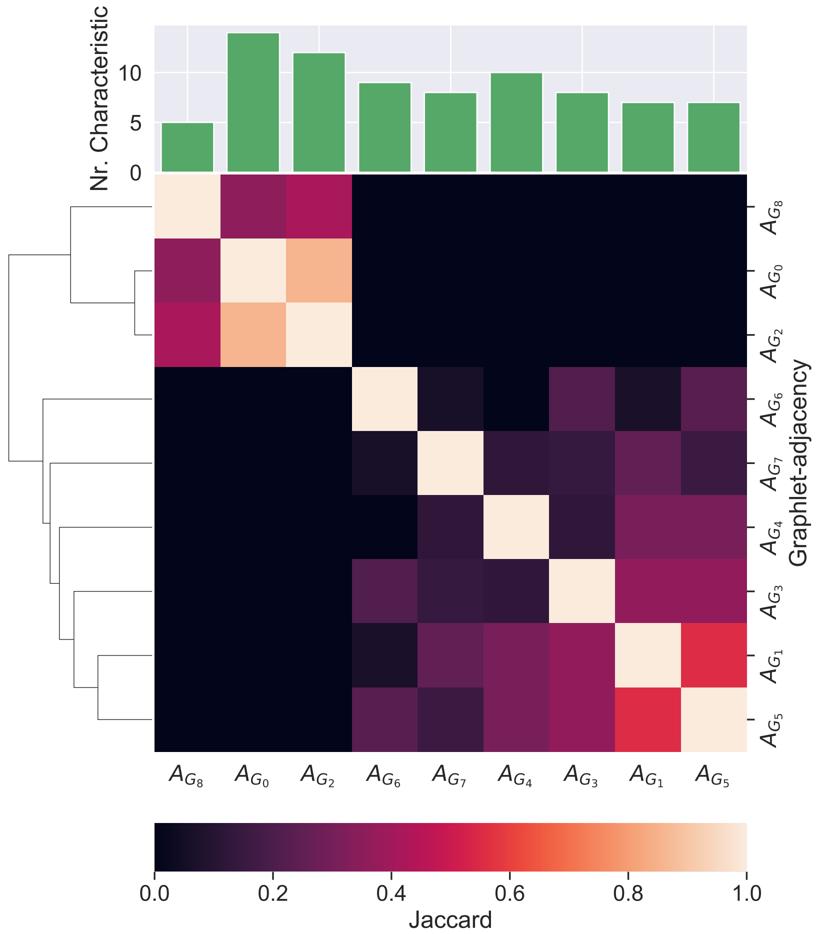

Supplementary Figure 23: **The number of pathways described by each graphlet adjacency and their overlap in the yeast COEX network.** A clustered heat map of the Jaccard similarity indices between the sets of pathways described by different graphlet adjacencies (x-axis). On top, a bar-chart indicating the number pathways described by each corresponding graphlet adjacency.

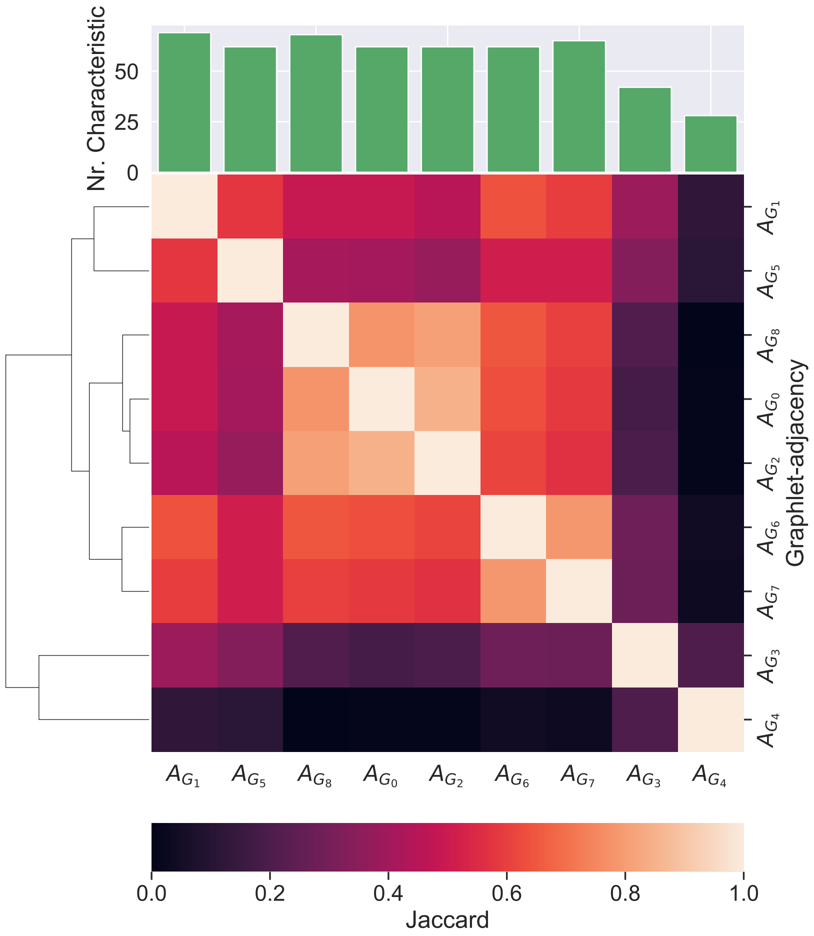

Supplementary Figure 24: **The number of pathways described by each graphlet adjacency and their overlap in the human COEX network.** A clustered heat map of the Jaccard similarity indices between the sets of pathways described by different graphlet adjacencies (x-axis). On top, a bar-chart indicating the number pathways described by each corresponding graphlet adjacency.

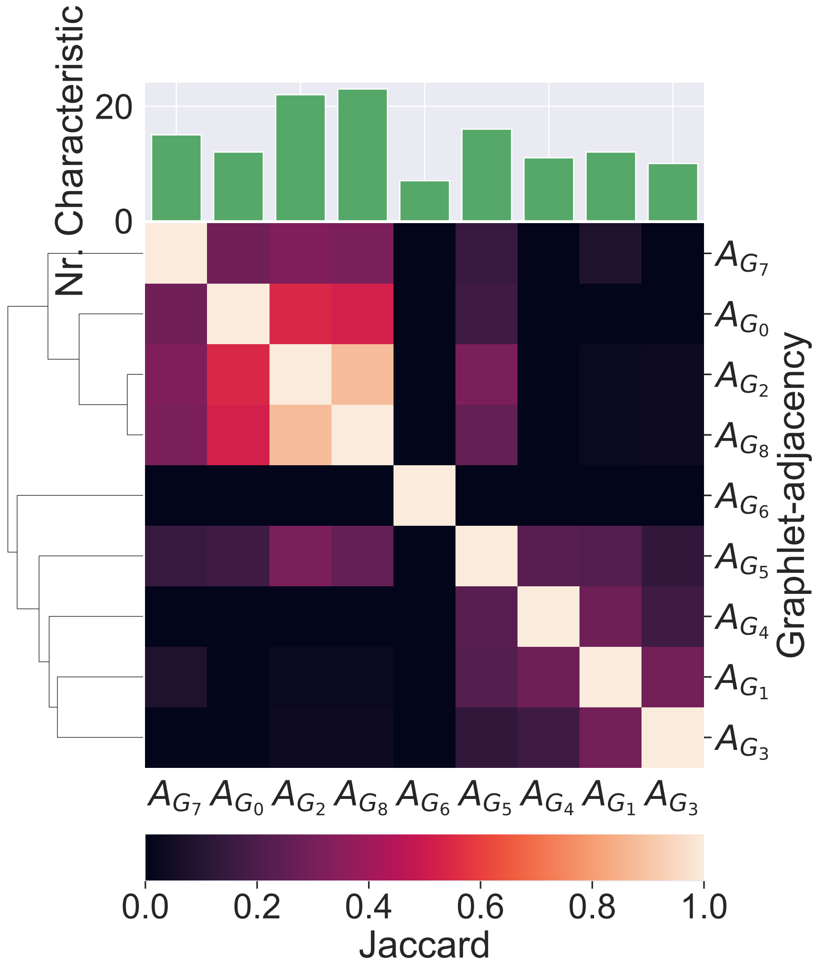

Supplementary Figure 25: **The number of pathways described by each graphlet adjacency and their overlap in the yeast GI network.** A clustered heat map of the Jaccard similarity indices between the sets of pathways described by different graphlet adjacencies (x-axis). On top, a bar-chart indicating the number pathways described by each corresponding graphlet adjacency.

4.3 Graphlet adjacencies describe complementary groups of functionally related pathways

In this section, we show that in each of our five molecular networks, each graphlet adjacency describes a set of pathways that is biologically functionally consistent in terms of the type of pathways they represent and the GO-terms in which they are enriched. Additionally, we show that the biological

function captured is specific to each graphlet adjacency.

To check if a given graphlet adjacency captures functionally similar pathways, we first annotate each pathway with its second level *ancestors*, i.e. the more generic pathways of which descendant, found one step away from the root nodes of the Reactome ontology (see Supplementary Section 2.2.1). Analogously, we annotate each pathway with the GO-terms in which its gene set is enriched (see Supplementary Section 2.2.2). Then, for each set of pathways described by a given graphlet adjacency, we perform pathway-set enrichment analysis (see Supplementary Section 2.2.3) to determine if the pathways in the set share biological function.

In the bar charts at the top of Supplementary Figures 26 to 30, we observe that in all five of our molecular networks, each graphlet adjacency describes pathways that are enriched in at least one ancestor annotation, GO-BP term, GO-CC term and GO-MF term. This means that in all five of our molecular networks, different graphlet adjacencies describe pathways that are functionally similar in terms of the types of ancestor annotations, GO-BP terms, GO-CC terms and GO-MF terms in which they are enriched. There is one exception to this conclusion in the yeast COEX network, where the set of pathways described by graphlet adjacency A_{G_1} is not enriched in any ancestor annotations, meaning these pathways are not statistically significantly similar in terms of the type of pathways they represent.

For our set of yeast molecular networks, in the heat maps presented in Supplementary Figures 26, 28 and 30, we generally find very low overlap between the functional annotations enriched in the pathway sets described by different graphlet adjacencies. This is true for all four of our different functional annotations (i.e. ancestor annotations, GO-BP terms, GO-CC terms and GO-MF terms). For yeast, the lowest overlap in terms of enriched functional annotations is achieved in the COEX network, where the average Jaccard index between the ancestors enriched in the pathways described by two different graphlet adjacencies 0.11. The highest overlap in terms of enriched functional annotations is achieved in the PPI network, where the average Jaccard index between the GO-MF enriched in the pathways described by two different graphlet adjacencies is 0.40.

For our set of human molecular networks, in the heat maps presented in Supplementary Figures 27 and 29, we generally find low overlap between the ancestor annotations, GO-CC terms and GO-BP terms enriched in the pathway sets described by different graphlet adjacencies. Of these three types of functional annotations, the lowest overlap is achieved in the PPI network, where the average Jaccard index between the ancestors enriched in the pathways described by two different graphlet adjacencies 0.17. The highest average overlap is achieved in the human PPI network, where the

average Jaccard index between the GO-BP terms enriched in the pathways described by two different graphlet adjacencies 0.45. Graphlet adjacencies describes pathways that are relatively similar in terms of molecular function in the human PPI network, as the average overlap between enriched GO-MF terms is 0.71.

We conclude that, apart from in terms of GO-MF terms in the human PPI network, pathways described by different graphlet adjacencies are functionally different in terms of the ancestor annotations, GO-BP terms, GO-CC terms and GO-MF terms in which they are enriched.

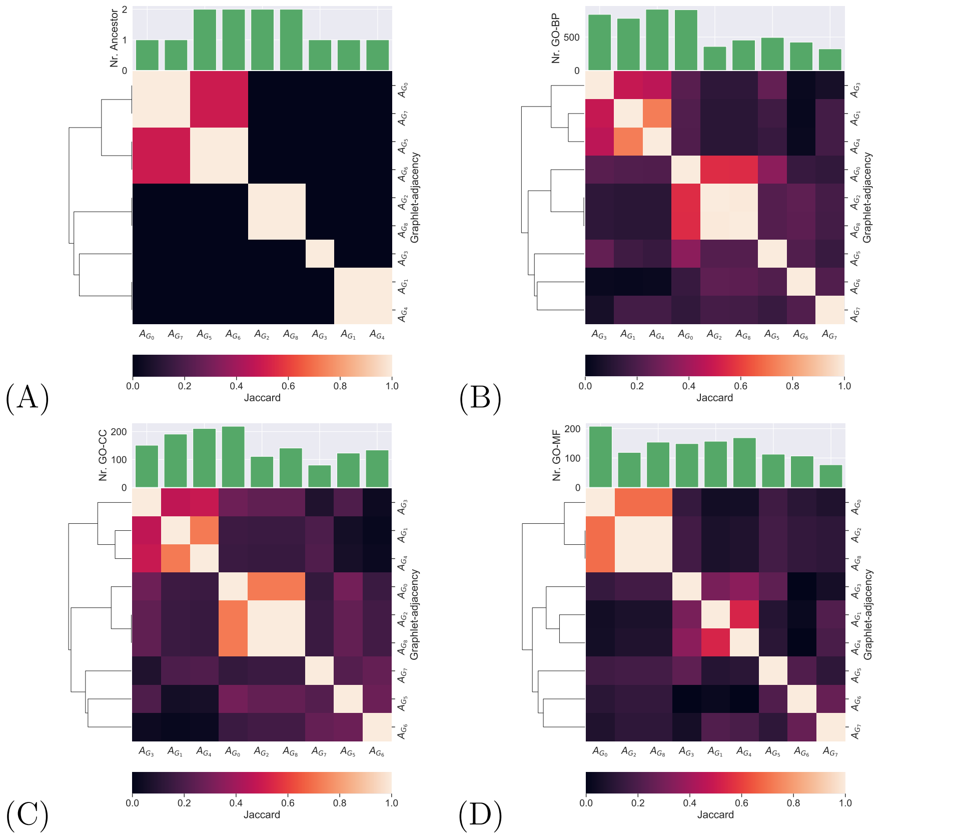

Supplementary Figure 26: **Biological similarity between pathways described by different graphlet adjacencies in the yeast PPI network.** Plots (A), (B), (C) and (D) respectively show a clustered heat map of the Jaccard similarity indices between the sets of ancestor annotations (A), GO-BP terms (B), GO-CC terms (C) and GO-MF terms (D), that are enriched in the sets of pathways described by different types of graphlet adjacencies (x-axis and y-axis). On top of each heat map, a bar-chart indicates the number of ancestor annotations (A), GO-BP terms (B), GO-CC terms (C) and GO-MF terms (D), that are enriched in the sets of pathways described by each corresponding graphlet adjacency (x-axis).

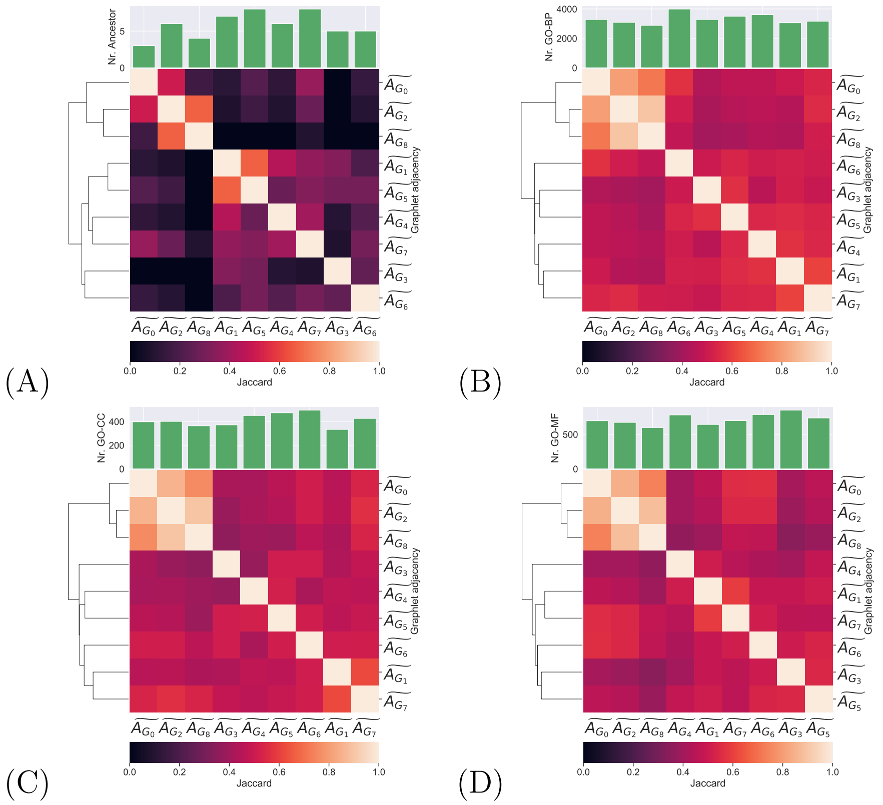

Supplementary Figure 27: **Biological similarity between pathways described by different graphlet adjacencies in the human PPI network.** Plots (A), (B), (C) and (D) respectively show a clustered heat map of the Jaccard similarity indices between the sets of ancestor annotations (A), GO-BP terms (B), GO-CC terms (C) and GO-MF terms (D), that are enriched in the sets of pathways described by different types of graphlet adjacencies (x-axis and y-axis). On top of each heat map, a bar-chart indicates the number of ancestor annotations (A), GO-BP terms (B), GO-CC terms (C) and GO-MF terms (D), that are enriched in the sets of pathways described by each corresponding graphlet adjacency (x-axis).

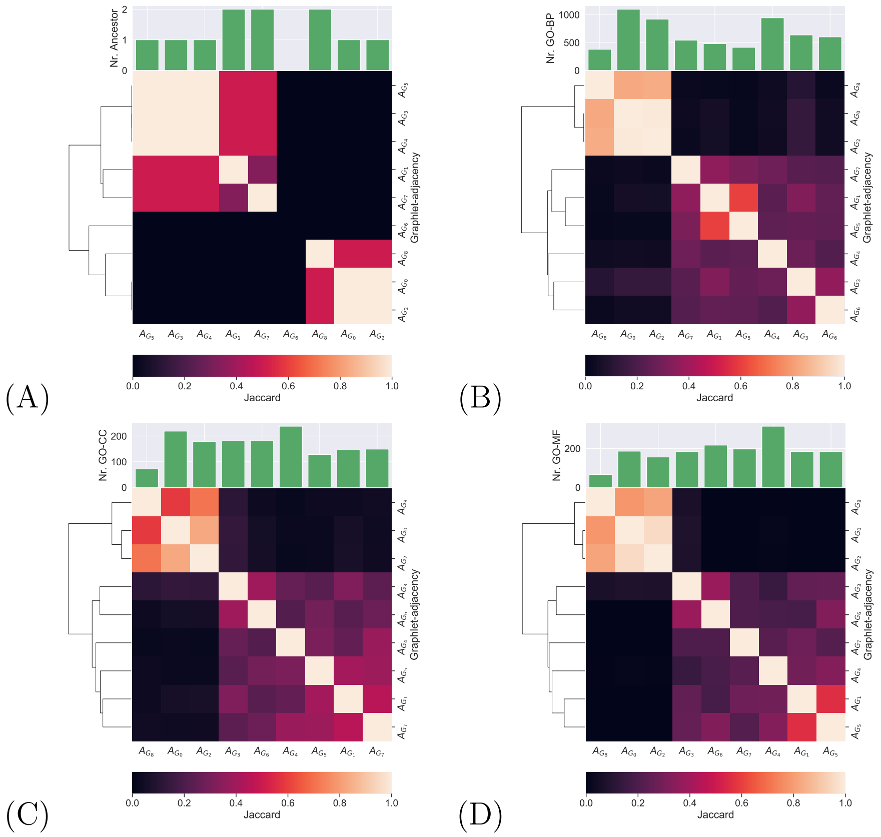

Supplementary Figure 28: **Biological similarity between pathways described by different graphlet adjacencies in the yeast COEX network.** Plots (A), (B), (C) and (D) respectively show a clustered heat map of the Jaccard similarity indices between the sets of ancestor annotations (A), GO-BP terms (B), GO-CC terms (C) and GO-MF terms (D), that are enriched in the sets of pathways described by different types of graphlet adjacencies (x-axis and y-axis). On top of each heat map, a bar-chart indicates the number of ancestor annotations (A), GO-BP terms (B), GO-CC terms (C) and GO-MF terms (D), that are enriched in the sets of pathways described by each corresponding graphlet adjacency (x-axis).

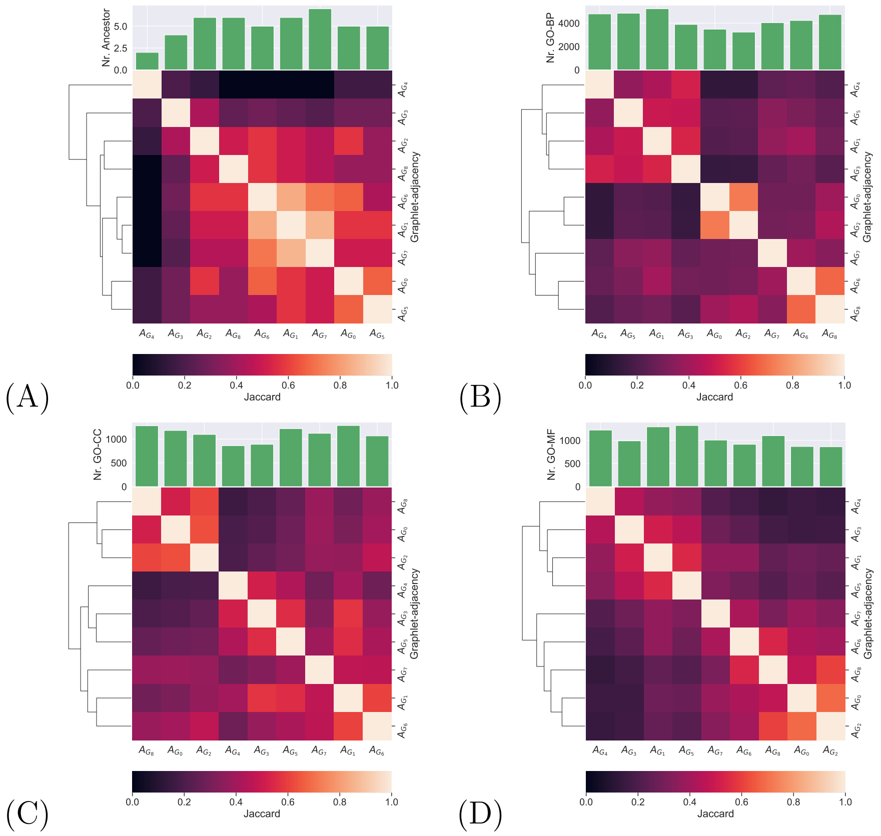

Supplementary Figure 29: **Biological similarity between pathways described by different graphlet adjacencies in the human COEX network.** Plots (A), (B), (C) and (D) respectively show a clustered heat map of the Jaccard similarity indices between the sets of ancestor annotations (A), GO-BP terms (B), GO-CC terms (C) and GO-MF terms (D), that are enriched in the sets of pathways described by different types of graphlet adjacencies (x-axis and y-axis). On top of each heat map, a bar-chart indicates the number of ancestor annotations (A), GO-BP terms (B), GO-CC terms (C) and GO-MF terms (D), that are enriched in the sets of pathways described by each corresponding graphlet adjacency (x-axis).

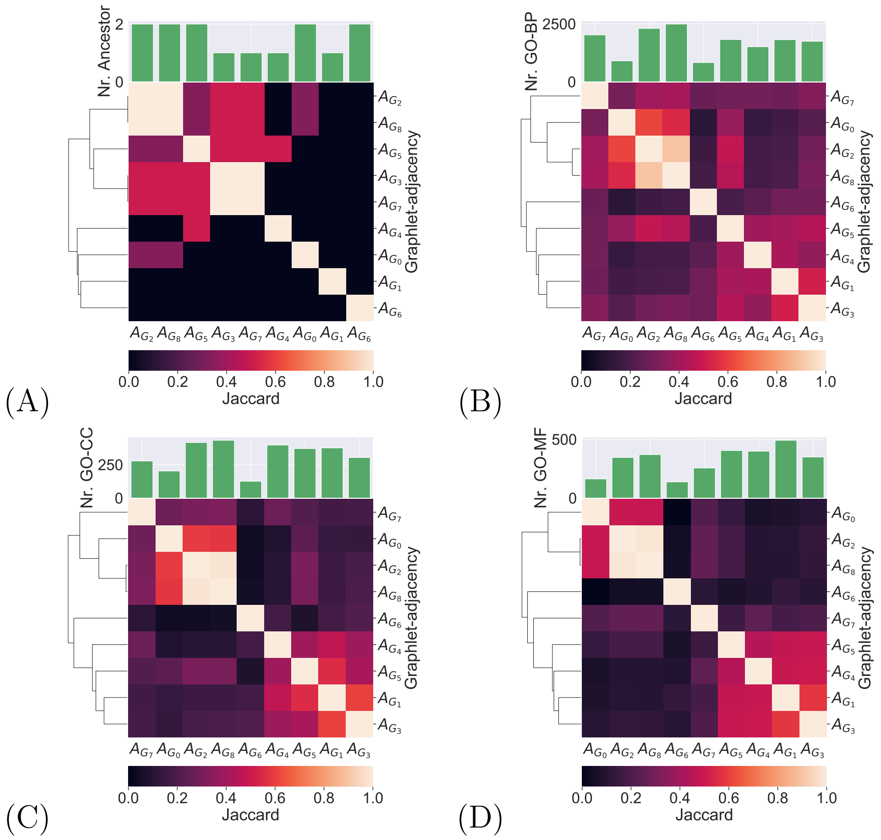

Supplementary Figure 30: **Biological similarity between pathways described by different graphlet adjacencies in the yeast COEX network.** Plots (A), (B), (C) and (D) respectively show a clustered heat map of the Jaccard similarity indices between the sets of ancestor annotations (A), GO-BP terms (B), GO-CC terms (C) and GO-MF terms (D), that are enriched in the sets of pathways described by different types of graphlet adjacencies (x-axis and y-axis). On top of each heat map, a bar-chart indicates the number of ancestor annotations (A), GO-BP terms (B), GO-CC terms (C) and GO-MF terms (D), that are enriched in the sets of pathways described by each corresponding graphlet adjacency (x-axis).

4.4 Pathways described by the same graphlet adjacency are topologically similar

Here we validate that the pathways that are described by the same graphlet adjacency are statistically significantly topologically similar.

To assess if the pathways described by the same graphlet adjacency are topologically similar, we compare the topological similarity between the path-

ways that are described by a given graphlet adjacency to all other pathways that are not described by it. We create two distance distributions: the topological distances between the pathways described by a given graphlet adjacency and a second distribution of distances between the pathways described by the given graphlet adjacency and the remaining pathways that are not (see Supplementary Figure 31 for the case of $\widetilde{A_{G_6}}$ in the human PPI network). Pathways described by the same graphlet adjacency are significantly topologically more similar to each other than to the remaining pathways, if the left-sided Wilcoxon-Mann-Whitney U-test (MWU) between the two distributions of distances is lower than or equal to 5% after application of the Benjamini and Hochberg (BH) correction for multiple hypothesis testing. As measures for network distance, we use GCD11, the degree distribution distance (DDD) and the clustering coefficient distance (CCD), defined in Supplementary Section 2.3. Results are summarised in Supplementary Figures 32 to 36.

We observe in all molecular networks that the pathways described by a given graphlet adjacency are topologically significantly similar to each other according to at least one network distance measure. For instance, in the yeast PPI network, pathways described by graphlet adjacency $\widetilde{A_{G_6}}$ are statistically significantly similar both in terms of their average clustering coefficient, degree distribution and graphlet correlations. Exceptions are the pathways described by $\widetilde{A_{G_3}}$ in the yeast PPI network, $\widetilde{A_{G_4}}$ in the yeast COEX network and the pathways described by $\widetilde{A_{G_1}}$ in the yeast GI network, of which we can not say they are statistically significantly topologically similar by any measure.

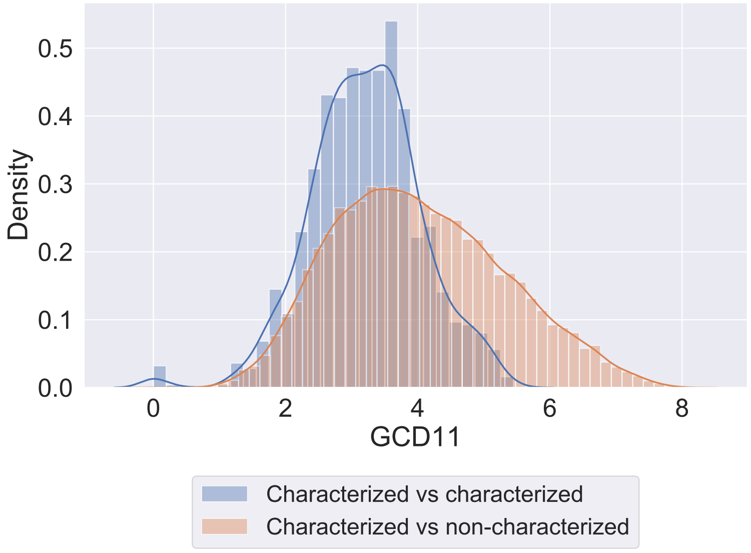

Supplementary Figure 31: **Pathways described by graphlet adjacency $\widetilde{A_{G_6}}$ in the human PPI network are statistically significantly topologically similar based on GCD11.** The GCD11 network distance distribution between the pathways described by graphlet adjacency $\widetilde{A_{G_6}}$ (blue) and the GCD11 distance distribution between the pathways described by graphlet adjacency $\widetilde{A_{G_6}}$ and the pathways not described by it. Applying a left-sided MWU-test, the adjusted p-value of 0.00 indicates that pathways described graphlet adjacency $\widetilde{A_{G_6}}$ are statistically significantly more similar to each other than to pathways not described by $\widetilde{A_{G_6}}$.

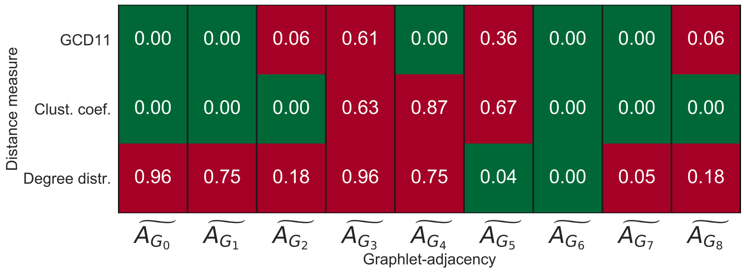

Supplementary Figure 32: **The statistical significance of the topological similarity of the pathways described by a given graphlet adjacency in the yeast PPI network.** A summary of adjusted p-values for the MWU tests measuring if pathways described by a given graphlet adjacency (x-axis) are statistically significantly topologically similar based on a given network-distance measures (y-axis).

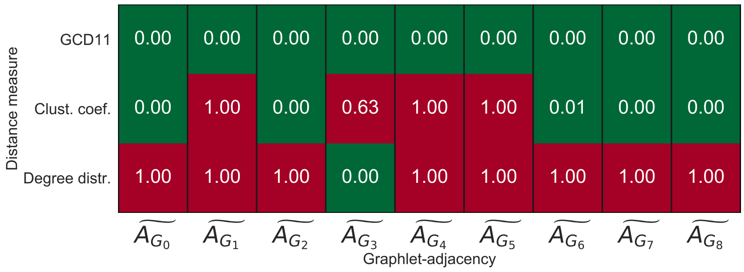

Supplementary Figure 33: **The statistical significance of the topological similarity of the pathways described by a given graphlet adjacency in the human PPI network.** A summary of adjusted p-values for the MWU tests measuring if pathways described by a given graphlet adjacency (x-axis) are statistically significantly topologically similar based on a given network-distance measures (y-axis).

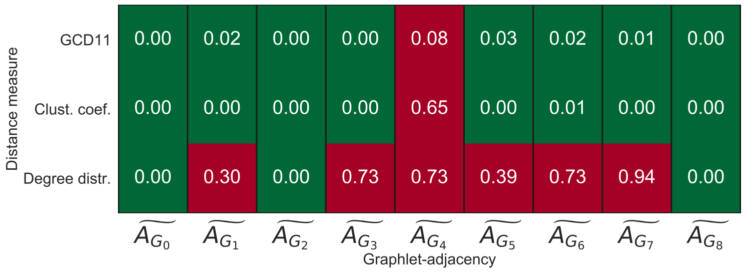

Supplementary Figure 34: **The statistical significance of the topological similarity of the pathways described by a given graphlet adjacency in the yeast COEX network.** A summary of adjusted p-values for the MWU tests measuring if pathways described by a given graphlet adjacency (x-axis) are statistically significantly topologically similar based on a given network-distance measures (y-axis).

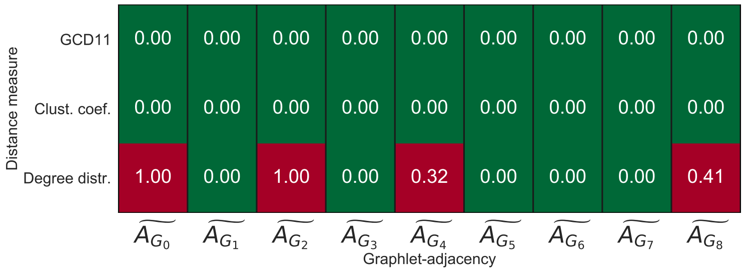

Supplementary Figure 35: **The statistical significance of the topological similarity of the pathways described by a given graphlet adjacency in the human COEX network.** A summary of adjusted p-values for the MWU tests measuring if pathways described by a given graphlet adjacency (x-axis) are statistically significantly topologically similar based on a given network-distance measures (y-axis).

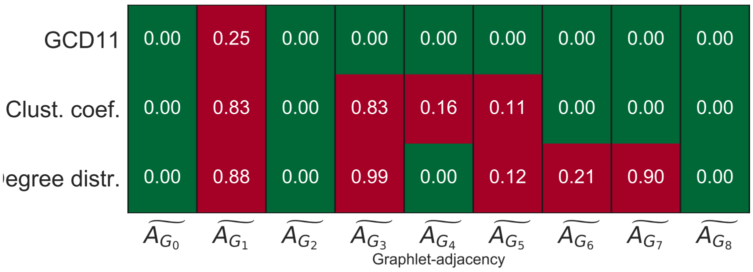

Supplementary Figure 36: **The statistical significance of the topological similarity of the pathways described by a given graphlet adjacency in the yeast GI network.** A summary of adjusted p-values for the MWU tests measuring if pathways described by a given graphlet adjacency (x-axis) are statistically significantly topologically similar based on a given network-distance measures (y-axis).

4.5 Linking pathways described by graphlet adjacencies to model networks

Having established that the pathways described by a given graphlet adjacency have statistically significantly similar topology, we investigate if this topology is similar to that of well-studied model networks. To that end, we compare the topology of each set of described pathways to that of well-studied model networks (see Supplementary Section 2.2). We create two distance distributions: the topological distances between the pathways described by a given graphlet adjacency, and a second distribution of the distances between the described pathways and randomly generated model networks. We consider a set of pathways described by the same graphlet adjacency to be indistinguishable of a given model network, if the two-sided MWU between the two distance distributions is lower than or equal to 5% after application of the Benjamini and Hochberg (BH) correction for multiple hypothesis testing.

In Figure 37-B, corresponding to the results in the human PPI network, we observe that pathways described graphlet adjacency A_{G_2} can not be distinguished from ER, ERDD SFGD and nPSO model networks. They are however, definitely not Geo, GeoGD or SF at the 5% significance level. We also observe that all sets of graphlet adjacency described pathways can not be topologically differentiated from nPSO model networks.

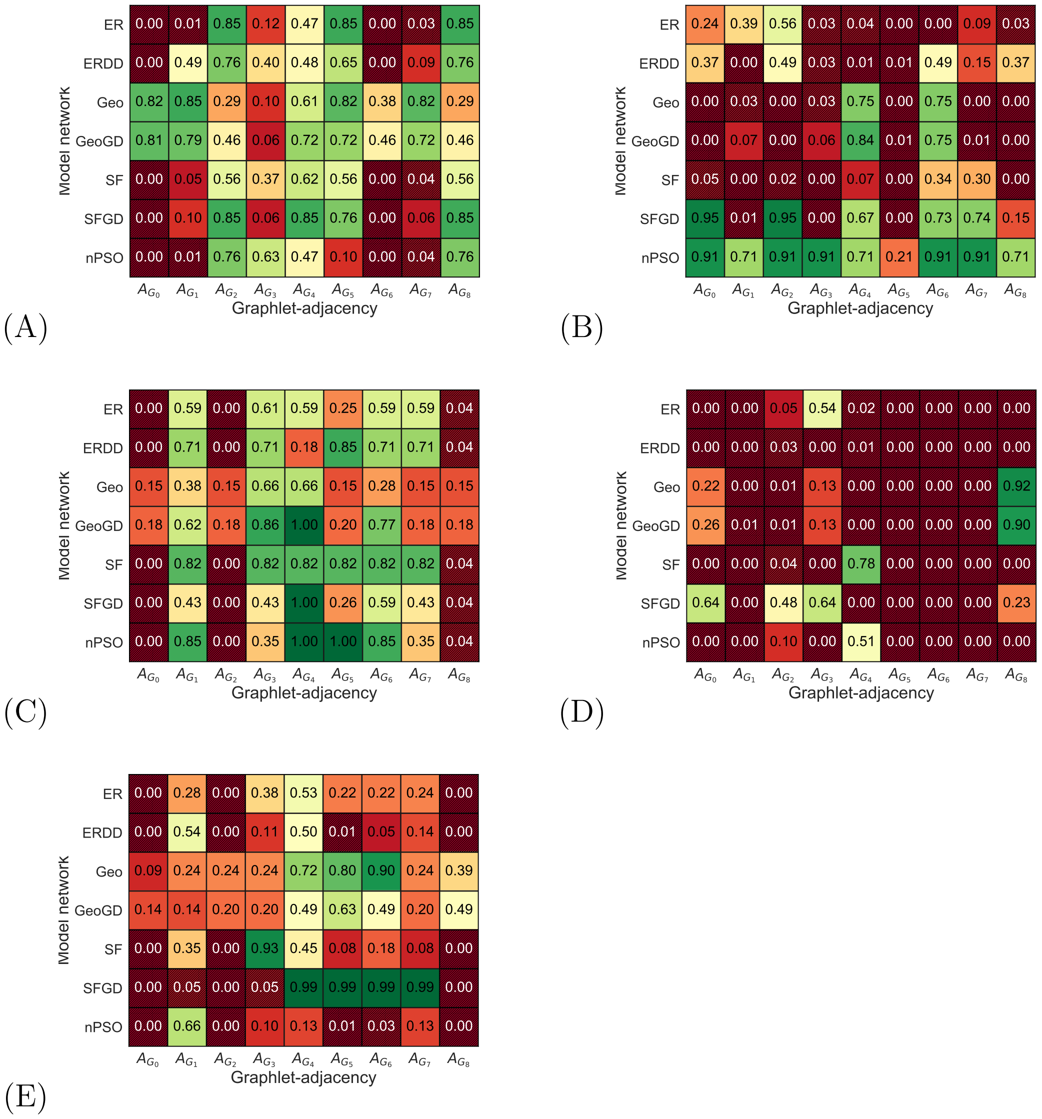

Supplementary Figure 37: **Linking the topology of described pathways to that of model networks.** The adjusted p-values for the MWU-test, testing if pathways described by a given type of graphlet adjacency (columns) can be distinguished from model networks (rows), based on GCD11 in: (A) the yeast PPI network, (B) the human PPI network, (C) the yeast COEX network, (D) the human COEX network and (E) the yeast GI network.

5 Graphlet eigencentralities capture complementary cancer mechanisms

5.1 Cancer related gene prediction accuracy

In experiment Section 3.2.1 of the main paper, we apply our graphlet centrality to predict cancer-related genes. We predict genes participating in a pathway to be cancer-related according to their pathway centrality. We consider the set of cancer driver genes listed by intOGen as our set of true positives (Gonzalez-Perez *et al.*, 2013). Results in the human PPI network and human COEX network are presented in Supplementary Figures 38 and 39, respectively.

In the PPI network, we observe that local and global graphlet eigencentralities approaches perform better than the expected AUC-ROC of 0.5 in case of random prediction accuracy, with median AUC-ROC scores over all pathways typically over 0.60, for each of the different underlying graphlets. Looking at AUC-PR performance to compare both approaches, we observe that our global graphlet eigencentrality approach based on graphlet adjacencies $\widetilde{A_{G_1}}$ and $\widetilde{A_{G_6}}$ achieves the highest overall prediction accuracy with an AUC-PR of 0.4 in both cases. Both when measuring prediction accuracy based on AUC-PR or AUC-ROC, global graphlet eigencentralities consistently outperform local graphlet eigencentralities, regardless of the underlying graphlet-adjacency considered. We hypothesise this is the case because pathways overlap and driver genes tend to the interactions between pathways. To support this hypothesis, we validate cancer driver genes occur in statistically significantly more pathways than non-driver genes. We apply a one sided Mann–Whitney U test in which we compare the distribution the number of pathways driver genes occur in, with the distribution of number of pathways non-driver genes occur in. Doing so, we achieve a significant p-value 5.19E−20. On average, cancer driver genes occur in 10.56 different pathways, whereas non-driver genes occur in only 6.07 different pathways.

We find similar results in the COEX network. In the COEX network, we observe that only global graphlet eigencentrality based on $\widetilde{A_{G_1}}$, $\widetilde{A_{G_3}}$, $\widetilde{A_{G_4}}$ and $\widetilde{A_{G_6}}$ performs better than the expected AUC-ROC of 0.5 in case of random prediction accuracy, achieving median AUC-ROC scores over all pathways of at least 0.60. For these graphlet adjacencies, we achieve a median AUC-PR 0.23 in all four cases. Again we observe that our global approach greatly outperforms our local approach in terms of median AUC-PR as well as median AUC-ROC. We validate that cancer driver genes occur in statistically significantly more pathways than non-driver genes. As before, we apply a one sided

Mann–Whitney U test in which we compare the distribution the number of pathways driver genes occur in, with the distribution of the number of pathways non-driver genes occur in. Doing so, we achieve a significant p-value 3.44E−13. On average, cancer driver genes occur in 6.31 different pathways, whereas non cancer driver genes occur in only 4.64 different pathways.

We conclude global eigencentrality is the best approach for finding pathways in which cancer-related genes play a central role.

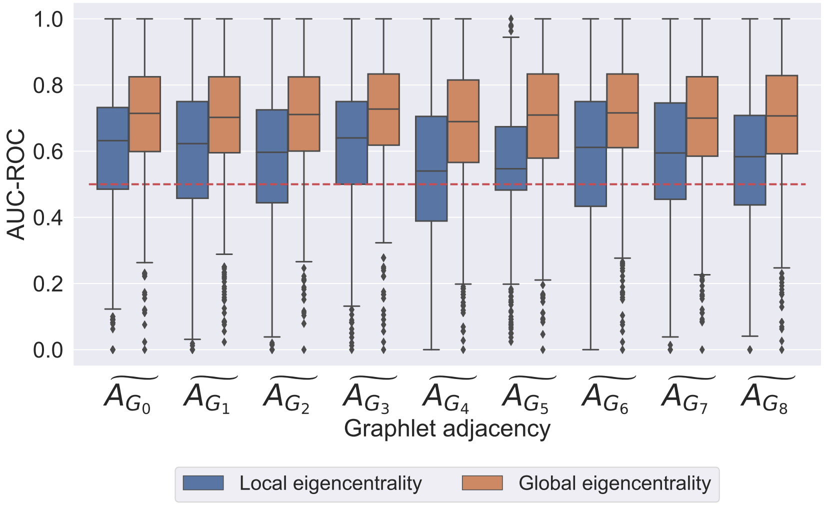

(A)

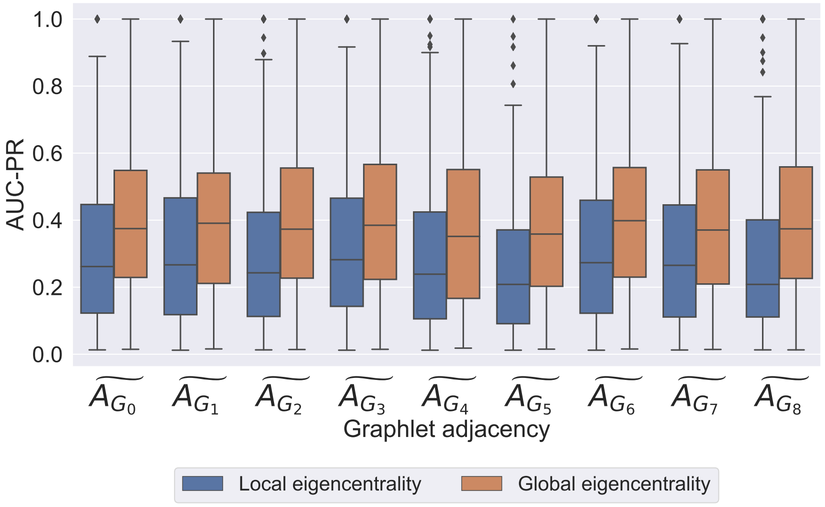

(B)

Supplementary Figure 38: **Cancer-related gene prediction accuracy** Panels (A) and (B) show the distribution of cancer-related gene prediction accuracies over all pathways as box plots, measured using AUC-ROC and AUC-PR respectively (y-axis), applying our local and global graphlet eigencentrality methods (colour, see legend), applied on different types of graphlet adjacencies (x-axis), in the human PPI network. In panel (A), a dashed red line at 0.5 indicates the expected AUC-ROC in case of random performance.

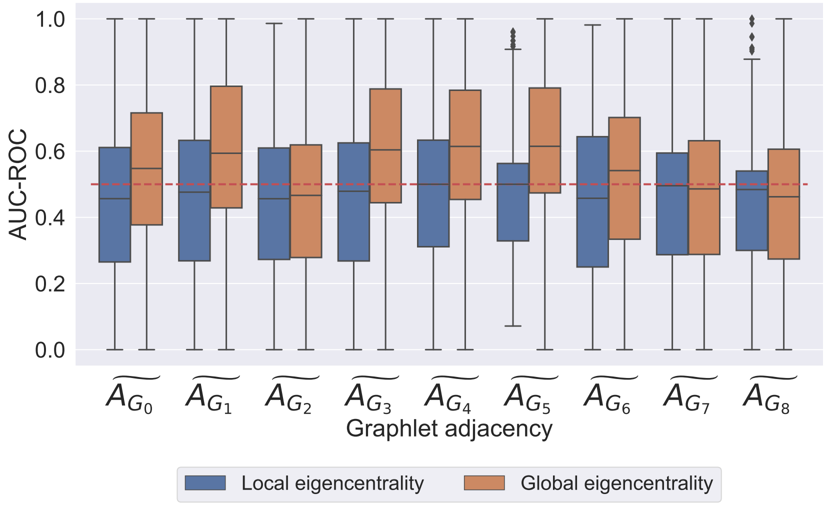

(A)

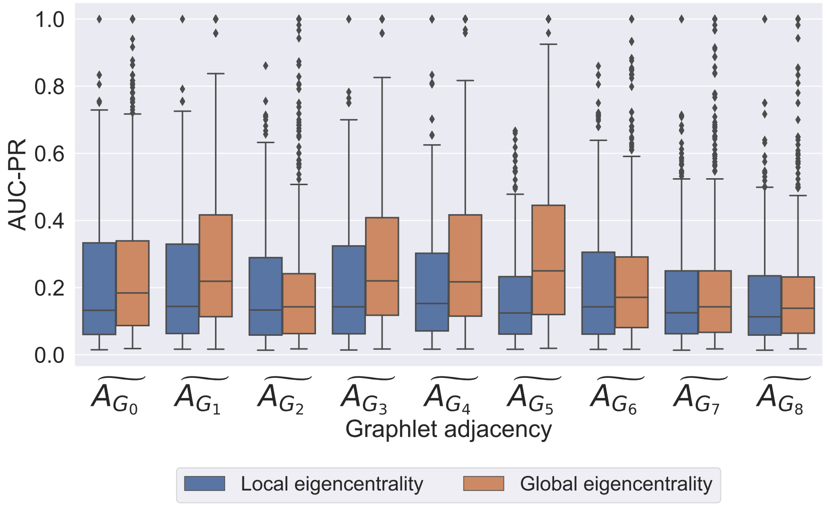

(B)

Supplementary Figure 39: **Cancer-related gene prediction accuracy** Panels (A) and (B) show the distribution of cancer-related gene prediction accuracies over all pathways as box plots, measured using AUC-ROC and AUC-PR respectively (y-axis), applying our local and global graphlet eigencentrality methods (colour, see legend), applied on different types of graphlet adjacencies (x-axis), in the human COEX network. In panel (A), a dashed red line at 0.5 indicates the expected AUC-ROC in case of random performance.

5.2 The number of cancer genes predicted and their overlap

In Section 3.2.2 of the main paper we compare the overlap between cancer-related genes found to be central in pathways described by central cancer genes, based on different graphlet-adjacencies. Here we show the results in both the human PPI network and the human COEX network. With an average Jaccard index of 0.30 in the human PPI network and 0.45 in the human COEX network, we conclude that different graphlet adjacencies describe the role in cancer of different sets of cancer related genes.

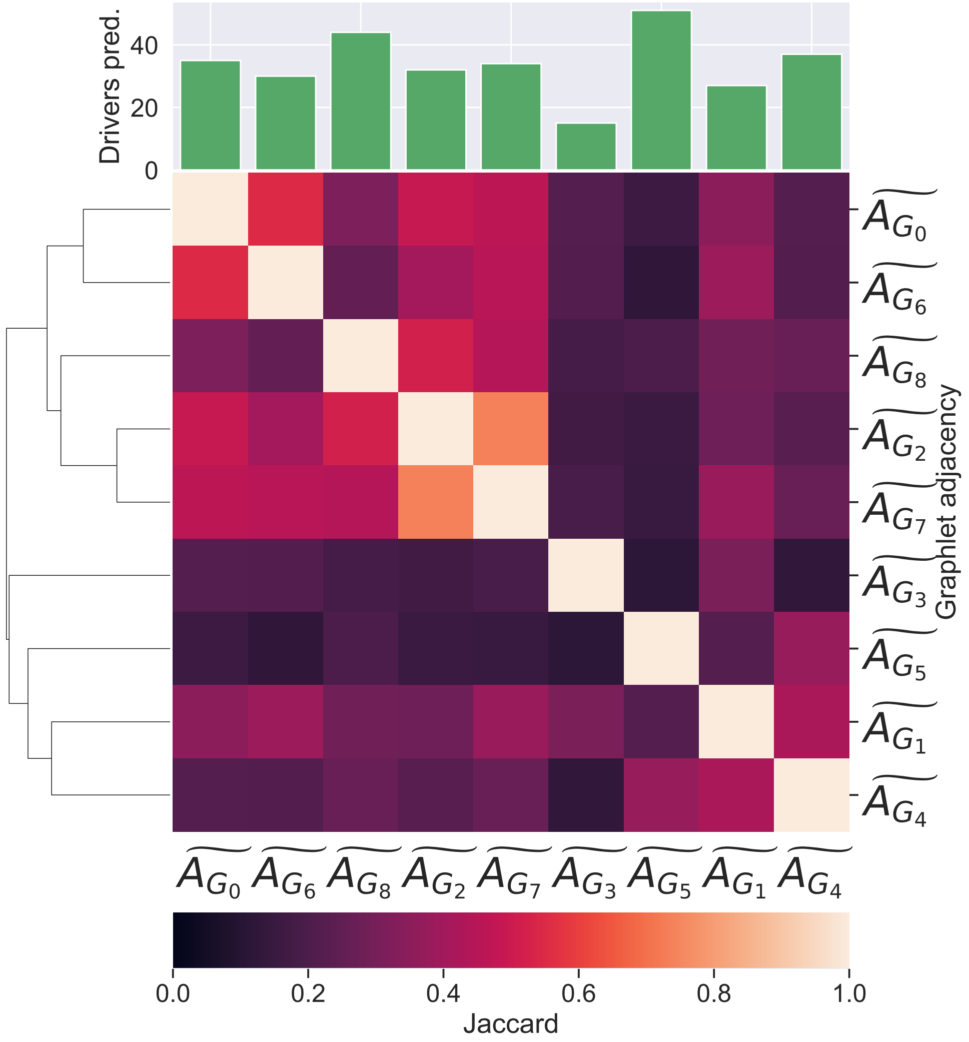

Supplementary Figure 40: **The overlap between correctly predicted cancer genes in pathways described by central cancer genes based on different graphlet adjacencies, in the human PPI network.** A clustered heat map of the Jaccard similarity indices between the sets of correctly predicted cancer genes found in pathways described by central driver genes based on different graphlet adjacencies. On top, a bar-chart indicating for each type of graphlet adjacency the number of correctly predicted genes.

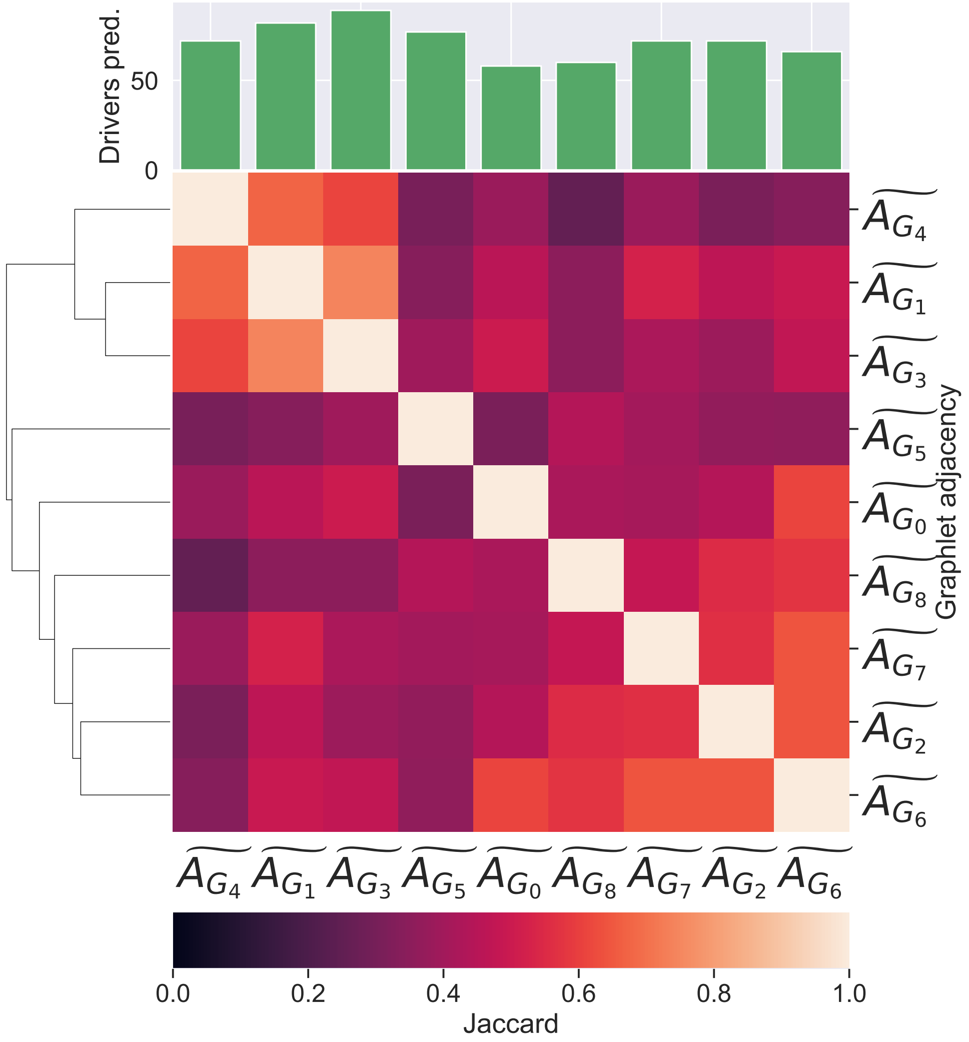

Supplementary Figure 41: **The overlap between correctly predicted cancer genes in pathways described by central cancer genes based on different graphlet adjacencies, in the human COEX network.** A clustered heat map of the Jaccard similarity indices between the sets of correctly predicted cancer genes found in pathways described by central driver genes based on different graphlet adjacencies. On top, a bar-chart indicating for each type of graphlet adjacency the number of correctly predicted genes.

References

Barabási, A. L. and Albert, R. (1999). Emergence of scaling in random networks. *Science*, **286**(5439), 509–512.

Erdős Paul and Rényi Alfréd, S. (1959). On random graphs. *Publicationes Mathematicae*, **6**, 290–297.

Gonzalez-Perez, A., Perez-Llamas, C., Deu-Pons, J., Tamborero, D., Schroeder, M. P., Jene-Sanz, A., Santos, A., and Lopez-Bigas, N. (2013). IntOGen-mutations identifies cancer drivers across tumor types. *Nature Methods*, **10**(11), 1081.

Janjić, V. and Pržulj, N. (2012). The core diseasome. *Molecular Biosystems*, **8**(10), 2614–2625.

Jeong, H., Mason, S. P., Barabási, A.-L., and Oltvai, Z. N. (2001). Lethality and centrality in protein networks. *Nature*, **411**(6833), 41–42.

Joy, M. P., Brock, A., Ingber, D. E., and Huang, S. (2005). High-betweenness proteins in the yeast protein interaction network. *Journal of Biomedicine and Biotechnology*, **2005**(2), 96.

Landherr, A., Friedl, B., and Heidemann, J. (2010). A critical review of centrality measures in social networks. *Business & Information Systems Engineering*, **2**(6), 371–385.

Milenković, T., Memišević, V., Bonato, A., and Pržulj, N. (2011). Dominating biological networks. *PLOS One*, **6**(8), e23016.

Muscoloni, A. and Cannistraci, C. V. (2018). A nonuniform popularity-similarity optimization (nPSO) model to efficiently generate realistic complex networks with communities. *New Journal of Physics*, **20**(5), 52002.

Newman, M. E. J. M. E. J. (2010). *Networks: An Introduction*. Oxford University Press.

Penrose, M. D. (2003). *Random Geometric Graphs*. Oxford University Press.

Pržulj, N. and Higham, D. J. (2006). Modelling protein–protein interaction networks via a stickiness index. *Journal of the Royal Society Interface*, **3**(10), 711–716.

Pržulj, N., Kuchaiev, O., Stevanović, A., and Hayes, W. (2010). Geometric evolutionary dynamics of protein interaction networks. In *Biocomputing 2010*, pages 178–189. World Scientific.

Windels, S. F. L., Malod-Dognin, N., and Pržulj, N. (2019). Graphlet Laplacians for topology-function and topology-disease relationships. *Bioinformatics*, **35**(24), 5226–5234.

Yaveroğlu, Ö. N., Malod-Dognin, N., Davis, D., Levnajic, Z., Janjic, V., Karapandza, R., Stojmirovic, A., and Pržulj, N. (2014). Revealing the hidden language of complex networks. *Scientific Reports*, **4**, 4547.

Yaveroğlu, Ö. N., Milenković, T., and Pržulj, N. (2015). Proper evaluation of alignment-free network comparison methods. *Bioinformatics*, **31**(16), 2697–2704.
